# BIBR1532 combined with radiotherapy induces ferroptosis in NSCLC cells and activates cGAS-STING pathway to promote anti-tumor immunity

**DOI:** 10.1186/s12967-024-05331-3

**Published:** 2024-05-30

**Authors:** Yawei Bao, Zhipeng Pan, Luqi Zhao, Jieping Qiu, Jingjing Cheng, Lei Liu, Dong Qian

**Affiliations:** 1 Department of Radiation Oncology, The First Affiliated Hospital of USTC, Division of Life Sciences and Medicine, University of Science and Technology of China, Hefei, Anhui 230001 China; 2 Core Facility Center, The First Affiliated Hospital of USTC, Division of Life Sciences and Medicine, University of Science and Technology of China, Hefei, Anhui China; 3 Department of Oncology, The Second Affiliated Hospital of Anhui Medical University, Hefei, Anhui 230000 China; 4 Pharmacological and Pharmaceutical Sciences, College of Pharmacy, University of Houston, Houston, TX 77204-5039 USA

**Keywords:** Anti-tumor immunity, cGAS-STING pathway, Ferroptosis, mtDNA release, Telomerase inhibitor

## Abstract

**Background:**

Telomerase, by safeguarding damaged telomeres and bolstering DNA damage repair, has the capacity to heighten the radioresistance of tumour cells. Thus, in turn, can compromise the efficacy of radiotherapy (RT) and radioimmunotherapy. Our previous studies have revealed that the highly selective telomerase inhibitor, BIBR1532, possesses the potential to enhance the radiosensitivity of Non-small cell lung cancer (NSCLC). In this study, we delve further into the impact of BIBR1532 on the immune activation induced by RT and elucidate the underlying mechanisms.

**Methods:**

Biological information analyses, immunofluorescence assays, western blot assays, flow cytometry analysis were conducted to elucidate the functions of the combination of BIBR1532 with radiotherapy in NSCLC. Intracellular levels of lipid peroxides, glutathione, malondialdehyde, and Fe^2+^ were measured as indicators of ferroptosis status. Both in vitro and in vivo studies were conducted to examine the antitumor effects.

**Results:**

Our findings indicate that the confluence of BIBR1532 with RT significantly augments the activation of the cGAS-STING pathway in both in vivo and in vitro settings, thereby fostering an effective anti-tumoral immune response. The effects can be ascribed to two key processes. Firstly, ionizing radiation, in precipitating DNA double-strand breaks (DSBs), prompts the release of tumour-derived double-stranded DNA (dsDNA) into the cytoplasm. Subsequently, BIBR1532 amplifies the activation of antigen-presenting cells by dsDNA post-RT and instigates the cGAS-STING pathway. Secondly, BIBR1532 enhances the ferroptosis response in NSCLC following RT, thereby promoting unrestrained lipid peroxidation and elevated levels of reactive oxygen species (ROS) within tumour cells. This ultimately leads to mitochondrial stress and the release of endogenous mitochondrial DNA (mtDNA) into the cytoplasm, thus facilitating the activation of the STING pathway and the induction of a type I interferon (IFN)-linked adaptive immune response.

**Conclusion:**

This study underscores the potential of BIBR1532 as an efficacious and safe radiosensitizer and radioimmunotherapy synergist, providing robust preclinical research evidence for the treatment of NSCLC.

**Supplementary Information:**

The online version contains supplementary material available at 10.1186/s12967-024-05331-3.

## Introduction

Non-small cell lung cancer (NSCLC) is a form of pulmonary malignancy originating from the bronchial mucosa, glands, and alveolar epithelium. Its elevated incidence and diminished survival rates categorize it among the foremost malignancies [1]. RT stands as the conventional and efficacious local treatment modality for all NSCLC stages. Furthermore, RT exerts a notable regulatory influence on the anti-tumour immune response [1–3]. RT resistance significantly contributes to tumour recurrence, yet clinical practice currently lacks safe and efficacious radiotherapy sensitization strategies [4–6], thereby constraining advancements in radiotherapy immunotherapy.

Telomeres, nucleoprotein complexes located at the extremities of linear eukaryotic chromosomes, serve to stabilise the structure of chromosome ends and avert fusion between ends. In so doing, they preserve chromosome integrity and genome stability [7]. Due to replication deficiencies, normal cells experience telomere shortening with each mitosis. When telomeres reach a critical length, they activate a DNA damage response (DDR), ultimately culminating in cellular senescence and apoptosis, a biological phenomenon referred to as the telomere crisis [8]. Most tumour cells navigate the telomere crisis by reactivating telomerase, enabling them to evade cell cycle checkpoints, thus attaining unbridled proliferation and immortality, including in lung cancer [9, 10]. Telomerase, a ribonucleoprotein reverse transcriptase comprising of a human ribonucleoprotein reverse transcriptase (hTERT) and RNA template, chiefly functions to maintain telomere length. In the majority of adult somatic cells, telomerase is inhibited [11]. An escalating body of research underscores that chemical agents which target telomeres or telomerase, in conjunction with ionising radiation, constitute a promising anti-tumour strategy [12, 13]. Drugs with direct effects on telomeres encompass telomere oligomers (T-oligos) and G-quadruplex stabilisers (telomere inhibitors, RHPS4), which could heighten RT sensitivity. Targeting telomerase activity alongside ionising radiation predominantly involves biological agents, including interfering RNA, such as nucleoside analogues (6-thio-dG, AZT), non-nucleoside inhibitors (BIBR1532), and antisense oligonucleotide inhibitors GRN163L [14–16]. Furthermore, immunotherapy with hTERT polypeptide GV1001, secreted polypeptide Vx-001, and dendritic cells GRVAC1 has entered clinical trials, demonstrating promising therapeutic outcomes in phase I and phase II clinical trials [17–19].

BIBR1532 represents a non-nucleoside synthetic small molecule compound that non-competitively binds to the active site on hTERT, thereby inhibiting telomerase activity. A prior investigation [20] conducted by our team has revealed that low concentrations of BIBR1532 can augment telomere dysfunction induced by ionizing radiation (IR). This augmentation is coupled with the inhibition of the ATM/CHK1/G2 checkpoint within the DNA damage repair pathway following radiotherapy, consequently enhancing the sensitivity of ionizing radiation to exterminate tumour cells. Research has elucidated that ionizing radiation can induce dsDNA breaks, causing damaged DNA to migrate from the nucleus to the cytoplasm. The dsDNA released into the cytoplasm is recognised by cyclic GMP-AMP synthase (cGAS), thereby activating the stimulator of interferon genes (STING) pathway in both tumour cells and DCs. The activated STING subsequently binds to tank-binding kinase 1 (TBK1), facilitating the phosphorylation of interferon regulatory factor 3 (IRF3) and up-regulation of IFN-β expression. This leads to the maturation of DCs and the development of robust anti-tumour immunity [21, 22]. Simultaneously, ionizing radiation can trigger ferroptosis in cells. On one front, fatty acid free radicals (PUFA•) generated post-IR irradiation rapidly with oxygen molecules, forming lipid peroxide free radicals (PUFA-OO•) and lipid hydroperoxides (PUFA-OOH) through the Fenton reaction. This cascade of events results in increased intracellular ROS and lipid peroxidation [23]. Furthermore, IR can induce the expression of SLC7A11 (cystine/ glutamate transporter, also known as xCT) and GPX4 (glutathione peroxidase 4). Their activation serves as a resistance mechanism against downstream signalling pathways, signifying an adaptive response to RT resistance. Following exposure to RT, the inactivation of GPX4 and SLC7A11 could lead to an increase in ROS levels in the cell. Subsequently, this process could trigger heightened lipid peroxidation, ultimately increasing cell death in response to radiotherapy and hence augmenting its efficacy. This forms the basis of numerous studies on ferroptosis drug treatment [24]. IR notably induces ferroptosis in tumour cells, at times surpassing the efficacy of many ferroptosis inducers (FINs). The synergy between IR and FINs elevates the expression of ferroptosis markers, including 4-HNE(4-hydroxynonenal) and PTGS2 (cyclooxygenase-2), further leading to a substantial radiosensitisation effect both in vitro and in vivo [25]. Consequently, the utilisation of FINs as a RT sensitizer in combination with radiotherapy emerges as a promising option for targeted ferroptosis in tumour treatment.

In this study, we have delved further into the facilitative role of BIBR1532 in enhancing the anti-tumour immune activation effect of RT. Our investigations have unveiled that the combination of BIBR1532 with radiotherapy impedes IR-induced DNA damage repair, resulting in a heightened frequency of dsDNA breaks. The dsDNA infiltrating the cytoplasm serves as a stimulus for cGAS induction, consequently inducing the activation of the STING pathway in DCs. Concurrently, BIBR1532 intensifies IR-induced ROS levels and lipid peroxidation, thereby inducing ferroptosis in tumour cells. Due to the absence of histone protection, mitochondria become more vulnerable to ROS [26, 27], initiating mtDNA stress and release into the cytoplasm. This prompts heightened sensitivity of cGAS to mtDNA, thereby further amplifying the potent anti-tumour immunity stemming from STING pathway activation. The dual activation of the cGAS-STING pathway by BIBR1532 presents an intriguing avenue of research, providing a novel direction for triggering ferroptosis and activating the STING pathway to foster anti-tumour immunity.

## Materials and methods

### Cell lines and cell culture

Human NSCLC cell lines, namely H460 and A549, were procured from the Shanghai Cell Bank of the Chinese Academy of Sciences (Shanghai, China). The LLC-OVA cell line, which expresses a single coding OVA SIINFEKL polypeptide and H-2Kbb2-M polypeptide, was graciously provided by Dr. Han at Tianjin Medical University Cancer Institute and Hospital (China). All cell lines were maintained in distinct media supplemented with 10% fetal bovine serum (FBS) (Gibco, USA). A549 and LLC-OVA cells were cultivated in DMEM (Invitrogen, USA) medium containing 10% FBS and 1% penicillin-streptomycin. The human NSCLC cell line H460 was cultured in RPMI-1640 medium supplemented with 10% FBS and 1% penicillin-streptomycin. The incubation was carried out in a 37 °C incubator with 5% CO_2_ and saturated humidity. All cell lines were authenticated by short tandem repeats profiling to ensure the absence of mycoplasma contamination.

BIBR1532 was procured from Selleck Chemicals (S1186, Houston, TX, USA). Cells were pre-treated with either BIBR1532 or dimethyl sulfoxide (DMSO) for 72 h, followed by exposure to radiation. with drug concentrations of A549 at 40 µM and H460 at 20 µM. Normal medium, devoid of BIBR1532, was substituted as per the experimental requirements, and all experiments were conducted following this protocol.

### Cell viability and cytotoxicity assay

Cell Counting Kit-8 (CCK-8, C0040, Beyotime, China) was employed to assess cell viability, and the Cytotoxicity LDH Assay Kit-WST® (CK12, Dojindo Laboratories Co., Ltd., Kumamoto, Japan) was utilised for cytotoxicity evaluation. A549 and H460 cells were plated in 96-well plates and incubated with various concentrations of BIBR1532. Cell assessments were carried out according to the manufacturer’s guidelines. Absorbance readings at 450 nm and 490 nm were recorded using a microplate reader (Bio-Rad, USA) at specified time points to evaluate cell growth and damage. Both experiments were conducted in triplicate.

### Colony formation assay

A549 and H460 cells were pre-treated with either DMSO or BIBR1532 for 72 h, then enumerated and seeded into 6-well plates at densities ranging from 100 to 2000 cells per well. Subsequent to adherence, the cells were exposed to radiation doses of 0, 2, 4, and 6 Gy, and were continuously cultured for 10–14 days without BIBR1532. The fresh medium replacement was performed every 3 days. Following colony formation, the cells were fixed with 4% paraformaldehyde for 15 min, and subsequently stained with 0.5% crystal violet at room temperature. Image capture was performed using a camera, with colony quantification including those consisting of at least 50 cells, and each group was assessed in triplicate wells.

### Cell migration and invasion assay

Digest and collect A549 and H460 cells, wash twice with PBS, resuspend in serum-free culture medium, and adjust cell concentration to 2 × 10^4^ cells/ml. Take a 24-well cell culture plate, add 600 µl of complete culture medium containing 10% FBS to each well, and gently place the transwell chamber (Corning, USA) of a PET membrane (8 μm) in it. Add 200 µl cell suspension containing BIBR1532 (H460 for 20 µM, A549 for 40 µM, according to the experimental plan) for each upper chamber. After cultured for 72 h, cells were exposed to a radiation dose of 4 Gy. After cultured for 24 h, discard the upper layer of culture medium and gently wipe off the upper layer of cells with a damp cotton swab. Subsequently, cells were fixed with 4% paraformaldehyde for 15 min, stained with 0.1% crystal violet for 20 min. Transmembrane cells from three randomly selected regions were photographed and counted using an inverted microscope (Olympus IX-71, Japan). Invasion experiment needs to evenly cover a layer of Matrix (BD Biosciences, USA) on the transwell PET membrane, and other experimental methods are consistent with the invasion assay.

### Animal model induction and treatment

All mice were procured from SiPeiFu (Beijing, China) and randomly allocated to their respective groups. Ethical oversight and approval for all animal experiments were obtained from the Ethics Committee of the First Affiliated Hospital of the University of Science and Technology of China. The mice were maintained under specific pathogen-free conditions, in adherence to national guidelines governing the use of research animals.

### Nude mouse model

A549 cells were subcutaneously inoculated into the proximal end of the right hind limb of female BALB/c nude mice (5 weeks old). Each mouse received a 100 µL PBS cell suspension containing 3 × 10^6^ cells. When the tumour volume reached 200 mm^3^, the mice were divided into four groups (*n* = 7). The experimental group received daily intraperitoneal injections of 1.5 mg/kg BIBR1532, while the control group received equivalent volumes of normal saline daily. On the fourth day, a photon beam linear accelerator (6MV-X-ray) (Varian, USA) was employed to administer a total dose of 10 Gy (2 Gy per day in 5 fractions) to the tumour site of the mice, using a custom fixture. Mouse body weights were measured every 3 days, and tumour dimensions (length and width) were assessed with a vernier caliper. Tumour volume was calculated using the formula V = length × width^2^/2. After one week of RT, the mice were euthanized, and tumour tissue was harvested for subsequent experiments.

### Syngeneic mouse model

Lewis lung cancer (LLC) has difficulty in immune response, and the LLC-OVA cell line expresses a single peptide encoding H-2Kbb2-M and OVA SIINFEKL peptide. Studies have shown that tumor APCs can uptake proteins (OVA) and dead cells, and can induce T cell proliferation in cells expressing OVA SIINFEKL peptide. Therefore, compared to LLC, LLC-OVA has stronger immunogenicity, and LLC-OVA cells were used for in vivo experiments. LLC-OVA cells were subcutaneously inoculated into the proximal end of the right hind limb of female C57BL/6J mice (5 weeks old). Each mouse received a 100 µL PBS cell suspension containing 2 × 10^6^ cells. When the tumour volume reached 100 mm^3^, the mice were grouped. Mouse body weights and tumour dimensions were measured every 3 days. The BIBR1532 group (1.5 mg/kg) received daily intraperitoneal injections until the end of RT. The total RT dose was 10 Gy (2 Gy per day in 5 fractions). The immune-checkpoint blockade treatment group received intraperitoneal injections of 250 µg/100 µL Anti-PD-L1 (BE0101, clone: 10 F.9G2 ™, BioXCell) every 3 days from the start of IR treatment. The control group received equal volume intraperitoneal injections of isotype control IgG (BE0090, clone: LTF-2, BioXCell). The C-176 group received daily intraperitoneal injections of 5 mg/kg (S6575, Selleck Chemicals) starting one week before RT and continued until the end of RT. Mice were euthanized when tumour volume reached 2000 mm^3^ or in the presence of severe complications, tumour tissue, peripheral blood and spleen samples were collected.

### Immunofluorescence (IF) assay

Cells were pre-treated with BIBR1532 or DMSO for 72 h and then seeded onto 24-well plates with a diameter of 14 mm (Biosharp, China) at a density of 2 × 10^4^ cells. Following exposure to a 4 Gy radiation dose, the cells were fixed with 4% paraformaldehyde for 15 min, permeabilized with 0.2% Triton X-100 for 10 min, and blocked with 10% bovine serum albumin (BSA) for 1 h. Subsequently, they were incubated with the primary antibody at 4°C overnight. The following day, cells were treated with a fluorescent secondary antibody for 1 h at room temperature, washed with phosphate buffer, and stained with 4’,6-diamidino-2-phenylindole (DAPI). Finally, the cell slides were inverted onto adhesive slides, and images were acquired using a laser confocal microscope (Zeiss LSM800, Germany). Comprehensive antibody information can be found in the Additional file 8: Table S1.

### PicoGreen staining

PicoGreen dsDNA Quantitation Reagent is an ultrasensitive fluorescent nucleic acid stain for quantitating double-stranded DNA (dsDNA) in solution. Cells were pre-treated with BIBR1532 or DMSO for 72 h and then placed in 24-well plates at a density of 1 × 10^4^ cells. The cells received a 4 Gy radiation dose. After 24 h, 3 µL/mL of PicoGreen (P11495, Thermo Scientific) dye was added, followed by incubation in a constant temperature incubator at 37 °C for 1 h. Following washing and fixation of the cells, the nuclei were re-stained using an anti-fluorescence quenching tablet containing DAPI and were observed under confocal microscopy, with images collected for analysis.

### Western blot analysis

Cells were lysed using a lysis buffer supplemented with a protein phosphatase inhibitor (P1260, Solarbio). Total protein was extracted, and the protein concentration was determined using a BCA protein concentration assay kit (23,227, Thermo Scientific). After separation by SDS-polyacrylamide gel electrophoresis, the proteins were transferred to a polyvinylidene fluoride membrane (Millipore, MA, USA). Subsequently, the membrane was blocked with 5% skimmed milk powder in Tween 20 detergent (TBST) at room temperature for 1 h. It was then incubated with the primary antibody at 4 °C overnight, followed by incubation with the secondary antibody at room temperature for 1 h. The ECL detection reagent (WP20005, Thermo Scientific) was utilised to detect the signal. Detailed antibody information can be found in the Additional file 8: Table S1.

### Reactive oxygen species (ROS) and lipid peroxidation detection

A549 and H460 cells were pre-treated and seeded in 6-well plates. After 24 h of exposure to a 4 Gy radiation dose, 10 µmol/L DCFH-DA (E004-1-1, Jiancheng Bioengineering Institute) was added and incubated in serum-free medium at 37 °C for 30 min in the dark. After washing, cells were resuspended in 500 µL PBS and analysed using flow cytometry (BD LSRFortessa, USA). To detect ROS in mitochondria, A549 and H460 cells were pre-treated and seeded in 24-well plates. After irradiation treatment, a 5 µM MitoSOX RED (40778ES50, Yeasen Biotechnology) mitochondrial superoxide red fluorescent probe was added. The probe was incubated at 37 °C for 30 min, washed, counterstained with DAPI, observed and photographed using confocal microscopy.

A549 and H460 cells were pre-treated and seeded in 6-well plates. After 48 h of irradiation treatment, 5 µM BODIPY 581/591 C11 dye (D3861, Invitrogen) was added and incubated at 37 °C for 30 min. The cell suspension was obtained after washing and digestion, and the level of lipid peroxidation was quantitatively assessed using flow cytometry.

### Transmission electron microscopy (TEM)

After subjecting the cells to different treatments, the supernatant was removed by centrifugation, and the cells were washed three times with 0.1 M phosphate buffer (PB, pH 7.4). A 1% agarose solution, pre-dissolved, was added, and the suspended cells were encased in agarose before solidification. The resin was fixed in 1% osmic acid (18,456, Ted Pella Inc.) at room temperature in the dark for 2 h. After rinsing, it was dehydrated at room temperature, embedded in acetone and 812 embedding agent (90529-77-4, SPI), and then transferred to an oven at 37 °C overnight. Subsequently, it was moved to an oven at 60 °C for polymerization for 48 h to create a resin block. Ultra-thin Sects. (60–80 nm) were generated using an ultra-thin sectioning machine (Leica UC7, Germany), and the sections were mounted on a 150-mesh copper grid. Images were observed and captured using a transmission electron microscope (HT7800, Hitachi, Japan).

### RNA extraction and RT‑qPCR

Total RNA was extracted from cells or tumour tissue using a cell/tissue total RNA kit (19221ES60, Yeasen Biotechnology), and the RNA concentration was determined using Nanodrop 2000 (Thermo Scientific, USA). Prime Script RT Master Mix (Takara, Japan) was used to synthesise cDNA. qRT-PCR was performed using TB Green Premix Ex Taq (Takara, Japan) in a real-time fluorescence quantitative PCR detection system (Roche, Switzerland). GAPDH was used as an internal reference, and the relative expression of genes was calculated using the 2^−ΔΔCt^ method. The primer details are provided in Additional file 8: Table S2.

### Glutathione (GSH) and malondialdehyde (MDA) detection

MDA is the final product of lipid peroxidation resulting from oxidative stress. The MDA detection kit (S0131S, Beyotime) utilises the colour reaction between MDA and thiobarbituric acid to produce red products, allowing the measurement of MDA levels in H460 and A549 cells following different treatments. Reduced glutathione (GSH) is the primary source of sulfhydryl groups in most living cells and plays a crucial role in maintaining the redox state of proteins. GSH and GSSG detection kits (S0053, Beyotime) were employed to determine the concentration of GSH in cells according to the manufacturer’s instructions after various treatments.

### Detection of intracellular Fe^2+^

The cells were treated and seeded in a confocal culture dish. After 24 h of irradiation with or without radiation, the intracellular content of Fe^2+^ in living cells was determined using the intracellular ferrous ion fluorescent probe Ferro Orange (F374, Dojindo). A Ferro Orange stock solution (1 mM) was prepared with DMSO and then diluted with HBSS (BL561A, Biosharp) to create a 1 µM working solution. The cells were incubated with the working solution at 37 °C for 30 min, and the images were observed and captured using confocal microscopy.

### Enzyme-linked immunosorbent assay (ELISA)

The chromogenic reaction involving biotin-labeled and peroxidase-labeled avidin, catalyzed by peroxidase, was employed. CXCL10 (DY466-05, R&D Systems) and IFN-β (MIFNB0, R&D Systems) were separately detected using a sandwich enzyme-linked immunosorbent assay kit. Tumour tissue samples were sectioned into small pieces, and a lysis buffer containing protease inhibitors was added. The samples were homogenized with a homogenizer. After centrifugation, the supernatant was collected and analysed in accordance with the manufacturer’s instructions.

### Flow cytometry analysis

Peripheral blood, spleen, and tumour tissue from mice were collected and processed into single-cell suspensions. Staining was carried out as per the antibody usage instructions (detailed antibody information was provided in the Additional file 8: Table S1). Nuclear expression staining was fixed and permeabilised using the True-Nuclear ^TM^ Transcription Factor Buffer Set (424,401, Biolegend, USA). Cytokine staining in the cytoplasm was treated with Wash Buffer (421,002, Biolegend), followed by fixation with intracellular staining fixation buffer (420,801, Biolegend) and permeabilisation. Antibody staining was performed at room temperature in the dark for 30 min, and the cells were washed and resuspended in PBS. Data were acquired using flow cytometry (BD FACSCanto II, USA) and analysed with FlowJo 10.8.1 software.

### Measurement of mitochondrial membrane potential (MMP)

The enhanced mitochondrial membrane potential detection kit (C2003S, Beyotime) was employed to assess changes in mitochondrial membrane potential. Healthy mitochondria exhibited red fluorescent polymers (J-aggregates). When the mitochondrial membrane potential was reduced, it existed in the form of green fluorescent monomers (J-monomer). The staining working solution was prepared by adding 1 mL of staining buffer to 5 µL JC-1 (200X). After thorough mixing with the cells, they were incubated at 37 °C for 20 min. Following washing, 2 mL of complete medium was added, and the cells were imaged using a fluorescence microscope.

### Immunohistochemistry (IHC) assay

Paraffin-embedded mouse tumour tissue samples were cut into 4 μm thick sections, dewaxed in xylene, and rehydrated in alcohol. After antigen retrieval, they were blocked with 3% hydrogen peroxide and goat serum to inhibit endogenous peroxidase activity. The sections were incubated with anti-GPX4 (dilution 1:200, ab125066, Abcam) and Ki-67 (5 µg/ml, ab15580, Abcam) at 4 °C. After secondary antibody staining, the sections were treated with a DAB kit (WE0323, Biorab), and then counterstained with modified Harris hematoxylin (G1150, Solarbio). Images of 3–10 different regions were captured using an optical microscope.

### RNA sequencing (RNA-seq) and quantitative proteomics (label-free)

Tumour tissue was collected, washed with sterile saline to remove blood stains, and non-tumour tissue was meticulously excised. Following water removal, the tissue was promptly placed in a frozen tube and submerged in liquid nitrogen. RNA sequencing and metabolomic analysis were conducted by Genesky Biotech Company (Shanghai, China). For RNA sequencing, the DESeq2 package was utilised to analyse the differentially expressed genes in the samples, with a screening threshold of | log_2_FC | ≥ 1.5 and an adjusted p-value < 0.05. In metabolomics analysis, liquid chromatography-mass spectrometry (LC-MS) technology, following sophisticated data analysis methods, was employed to quantify protein enzymatic peptides. A Peptide FDR ≤ 0.05 was used as the screening criterion based on peak intensity or peak area of related peptides in the first-order mass spectrometry. All differentially expressed metabolites were chosen for heat map and enrichment analysis.

### Statistical analysis

All data are presented as mean ± standard deviation. A two-tailed unpaired t-test was employed for comparisons between two groups, while one-way analysis of variance and Tukey’s multiple comparison test were used for the analysis of more than two groups. A *P* < 0.05 was considered statistically significant. Statistical analyses and graphical representations were performed using GraphPad Prism 9.0 software (GraphPad Software Inc., USA).

## Results

### BIBR1532 enhances radiosensitivity of NSCLC in vitro and in vivo

**Fig. 1:**
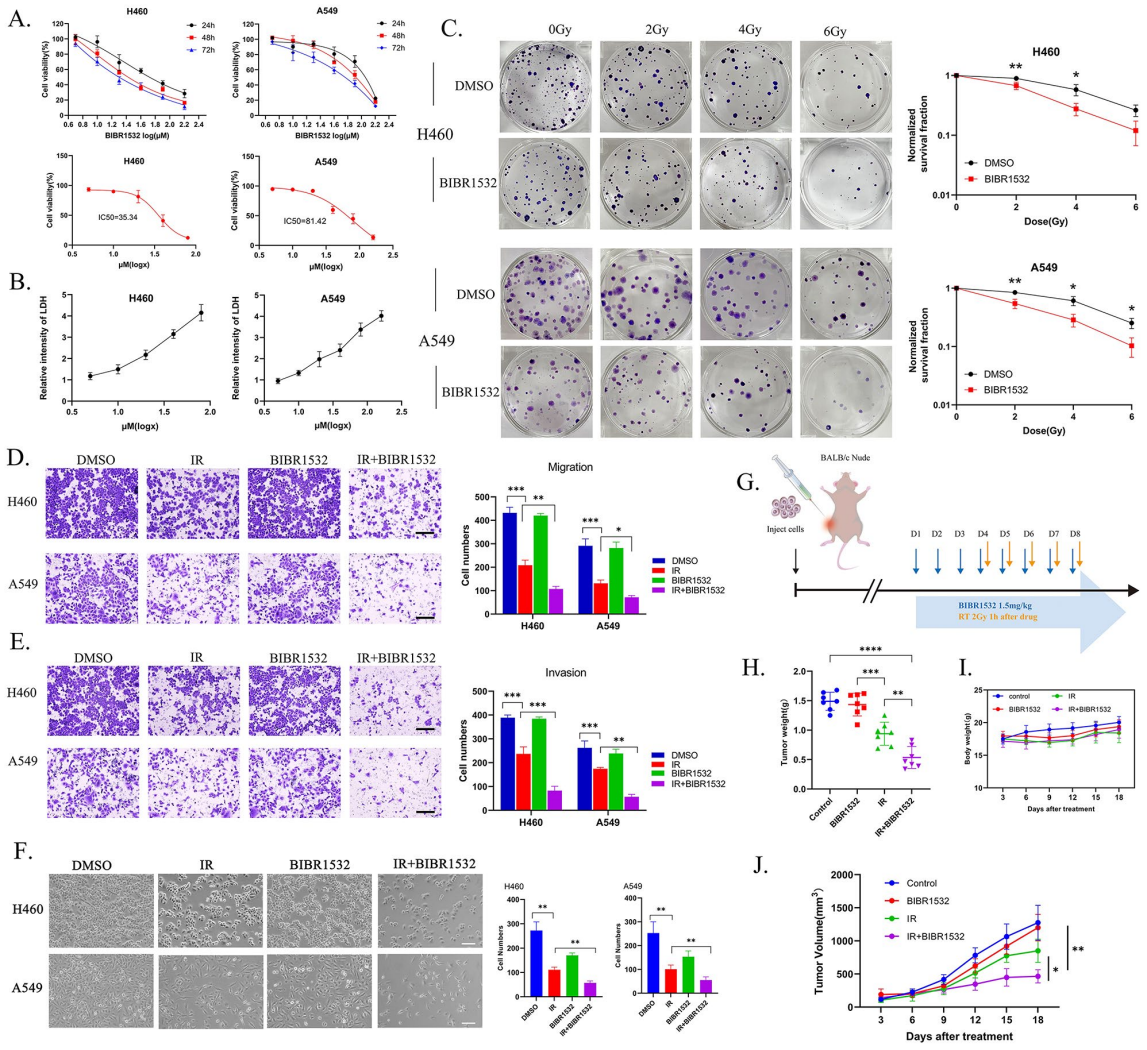
BIBR1532 can enhance the radiosensitivity of NSCLC in vitro and in vivo. **A** Cell viability was assessed using the CCK-8 method, and the IC50 value was determined after 72 h of co-incubation with H460 and A549 cells. **B** Following a 72-hour incubation with different concentrations of BIBR1532, LDH concentration was assessed, and the cellular damage rate was calculated. **C** The representative images of H460 and A549 cell colonies formed after irradiation with 0, 2, 4, and 6 Gy were utilized, and the cell survival curve was fitted using the linear-quadratic model. Transwell assays were employed to assess cell migration **D** and invasion **E** of NSCLC cells treated with BIBR1532 alone or in combination with IR. Scale bar = 200 μm. Cell counting was performed using Image J software. The collected data was then imported into GraphPad Prism software for statistical analysis using one-way ANOVA with Tukey’s multiple comparisons. The data was represented as mean ± SD, and each experiment was independently repeated three times. **F** H460 and A549 cells were treated with BIBR1532 alone or in combination with IR for 72 h. The morphology (left) and quantification (right) of H460 and A549 cells were observed under a light microscope (Scale bar = 50 μm). **G** BALB/c Nude mice were inoculated with A549 xenograft tumours. When the tumour volume reached 200 mm^3^, they were randomly divided into the control group, BIBR1532 group, IR group, and IR + BIBR1532 group for treatment (*n* = 7). **H-J** Tumour weight, mouse body weight, and tumour volume are depicted in the figure. All cells were pretreated with BIBR1532 for 72 h, and each experiment was repeated three times. The data is presented as mean ± standard deviation. *P < 0.05, **P < 0.01, ***P < 0.001, ****P < 0.0001, ns = not significant

A549 and H460 cells were exposed to various concentrations of BIBR1532 for 24 h, 48 h, and 72 h. The impact of BIBR1532 on the growth of NSCLC cells was assessed using a CCK-8. The findings revealed that a 72-hour exposure to BIBR1532 had a more pronounced inhibitory effect on cell viability compared to 24 h and 48 h. The IC50 values were 81.42 µM for A549 and 35.34 µM for H460 **(**Fig. 1A**)**. These results indicated that BIBR1532 exerted a dose- and time-dependent inhibition of cell viability. When the cell membrane is compromised, cytoplasmic lactate dehydrogenase (LDH) is released into the culture medium. Measurement of LDH levels in the culture medium serves as an indicator for assessing cell damage. Our results showed that higher BIBR1532 concentrations correlated with increased LDH release **(**Fig. 1B**)**, signifying greater cellular damage. Subsequently, we selected a 3-day drug exposure period for colony formation experiments. The addition of BIBR1532 significantly suppressed the colony formation capability of NSCLC cells compared to the IR-only group, following survival normalization after exposure to 0 Gy **(**Fig. 1C**)**. Additionally, the combination of BIBR1532 and IR notably inhibited the migration and invasion of NSCLC cells, whereas BIBR1532 alone had no effect on the cells **(**Fig. 1D, E**)**. Furthermore, we observed and documented changes in cell morphology and quantity for both cell lines after various treatments under an optical microscope **(**Fig. 1F**)**. The introduction of BIBR1532 in combination with RT did not substantially alter cell morphology but did lead to a significant reduction in cell number.

To investigate the impact of BIBR1532 on radiosensitivity in vivo, the experimental procedure is depicted in **(**Fig. 1G**)**. Results of our tumour-bearing nude mice experiment showed that the administration of BIBR1532 alone did not alter the tumorigenicity of H460 cells. In comparison to RT alone, the combination of RT and BIBR1532 markedly delayed tumour growth without affecting the mice’s body weight **(**Fig. 1H-J**)**. These findings align with the in vitro results, underscoring BIBR1532’s ability to enhance the radiosensitivity of NSCLC both in vitro and in vivo.

### BIBR1532 impedes IR-induced DNA double-strand break repair and augments the STING pathway

**Fig. 2:**
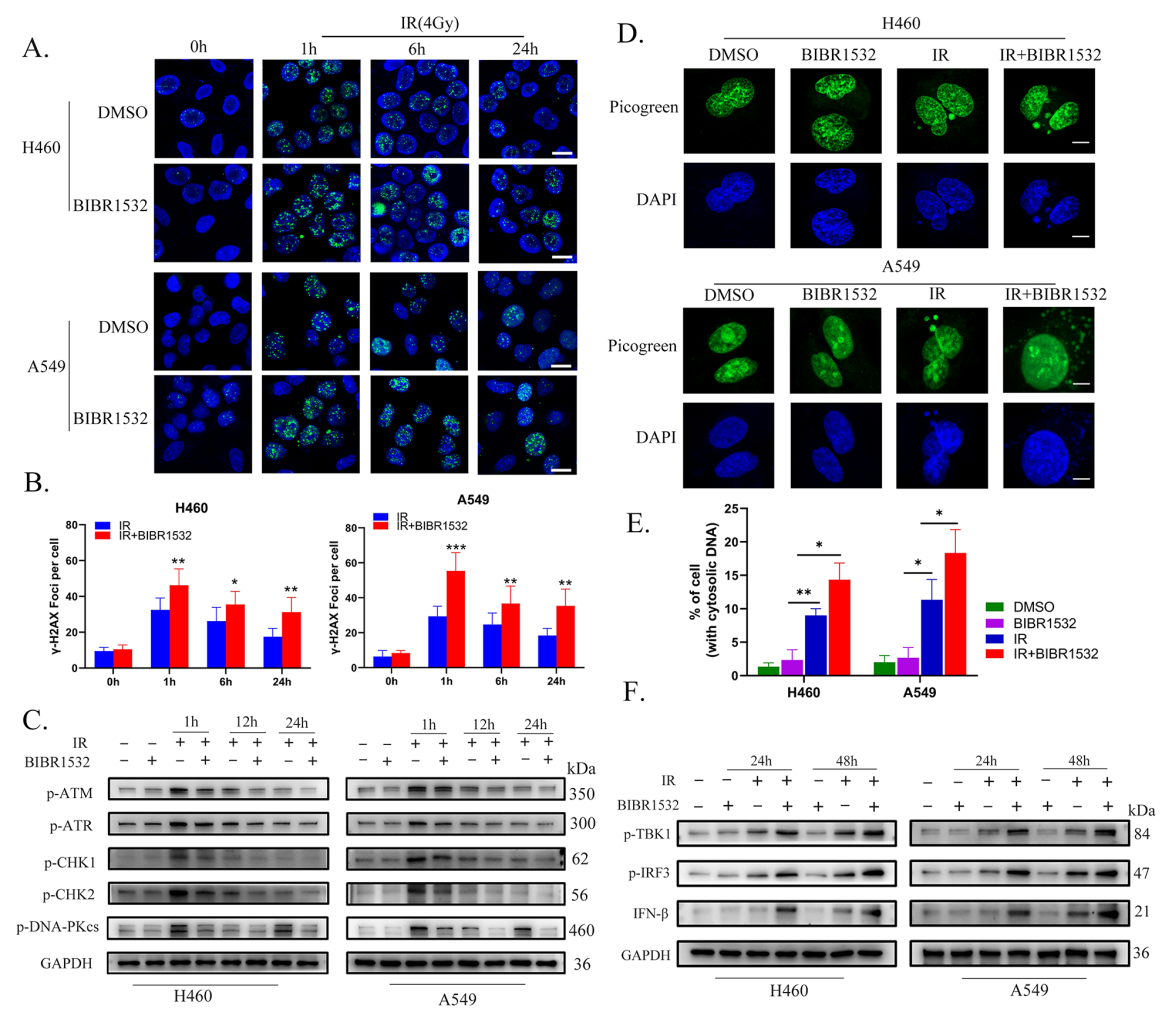
BIBR1532 inhibits the DDR after IR and enhances the activation of STING pathway. **A, B** Cells were pre-treated with or without BIBR1532, and γ-H2AX foci in H460 and A549 cells were assessed at specified time points following irradiation (4 Gy). The γ-H2AX foci were marked in green, and the nuclei were restained with DAPI (blue). The data represents the average number of foci from 10 randomly selected high-magnification fields (20X). **C** Expression levels of phosphorylated ATM, ATR, CHK1, CHK2, and DNA-PKcs at different time points after irradiation (4 Gy) were quantitatively assessed via Western blot and normalized to GAPDH. The results are based on *n* = 3 independent replicates. **D, E** Typical images of PicoGreen staining and quantitative analysis. Cells containing cytoplasmic DNA were quantified. Scale bar = 10 μm. **F** Western blot results demonstrated that BIBR1532 combined with IR increased the expression of p-TBK1, p-IRF3, and IFN-β. All data are presented as mean ± SD, and each experiment was independently repeated three times. * *p* < 0.05, * * *p* < 0.01, ***P < 0.001

The most severe form of DNA damage induced by ionizing radiation is the formation of DNA double-strand breaks (DSBs). In our investigation, we aimed to ascertain whether BIBR1532 exerts an influence on the repair of IR-induced DNA damage. One hour post-irradiation, both H460 and A549 cells displayed a greater number of γ-H_2_AX foci in the combined treatment group than in cells exposed to IR alone **(**Fig. 2A, B**)**. Notably, at the 24-hour mark following IR, residual γ-H_2_AX foci persisted in the combined treatment group in comparison to cells subjected to IR alone, indicating that potentially lethal DSBs remained unrepaired. Furthermore, we assessed the impact of BIBR1532 on key molecules involved in DSB repair, including ATM, ATR, CHK1, CHK2, and DNA-PKcs. When combined with IR, BIBR1532 reduced the expression of phosphorylated ATM, ATR, CHK1, CHK2, and DNA-PKcs compared to IR treatment alone (Fig. 2C and Additional file 1: Fig. S1A-E). The impairment in DNA DSB repair leads to the accumulation of damaged DNA within the nucleus. When this damaged DNA translocates into the cytoplasm, it can trigger the cytoplasmic DNA receptor cGAS, thereby activating STING and its downstream proteins [28].

As anticipated, BIBR1532 induced the accumulation of cytoplasmic dsDNA during IR exposure (Fig. 2D, E). Additionally, the expression levels of downstream proteins associated with the cGAS-STING pathway, such as phosphorylated TBK1, IRF3, and IFN-β, were significantly elevated. Both phosphorylated TBK1 and IRF3, crucial components of the STING pathway, displayed increased expression levels upon combined IR and BIBR1532 treatment (Fig. 2F and Additional file 1: Fig. S1F-H). These findings collectively demonstrate that BIBR1532 disrupts the repair of IR-induced DSBs, elevates the accumulation of cytoplasmic dsDNA, and amplifies the activation of the STING pathway as a response to IR treatment.

### BIBR1532 enhances IR-induced ROS and ferroptosis

**Fig. 3:**
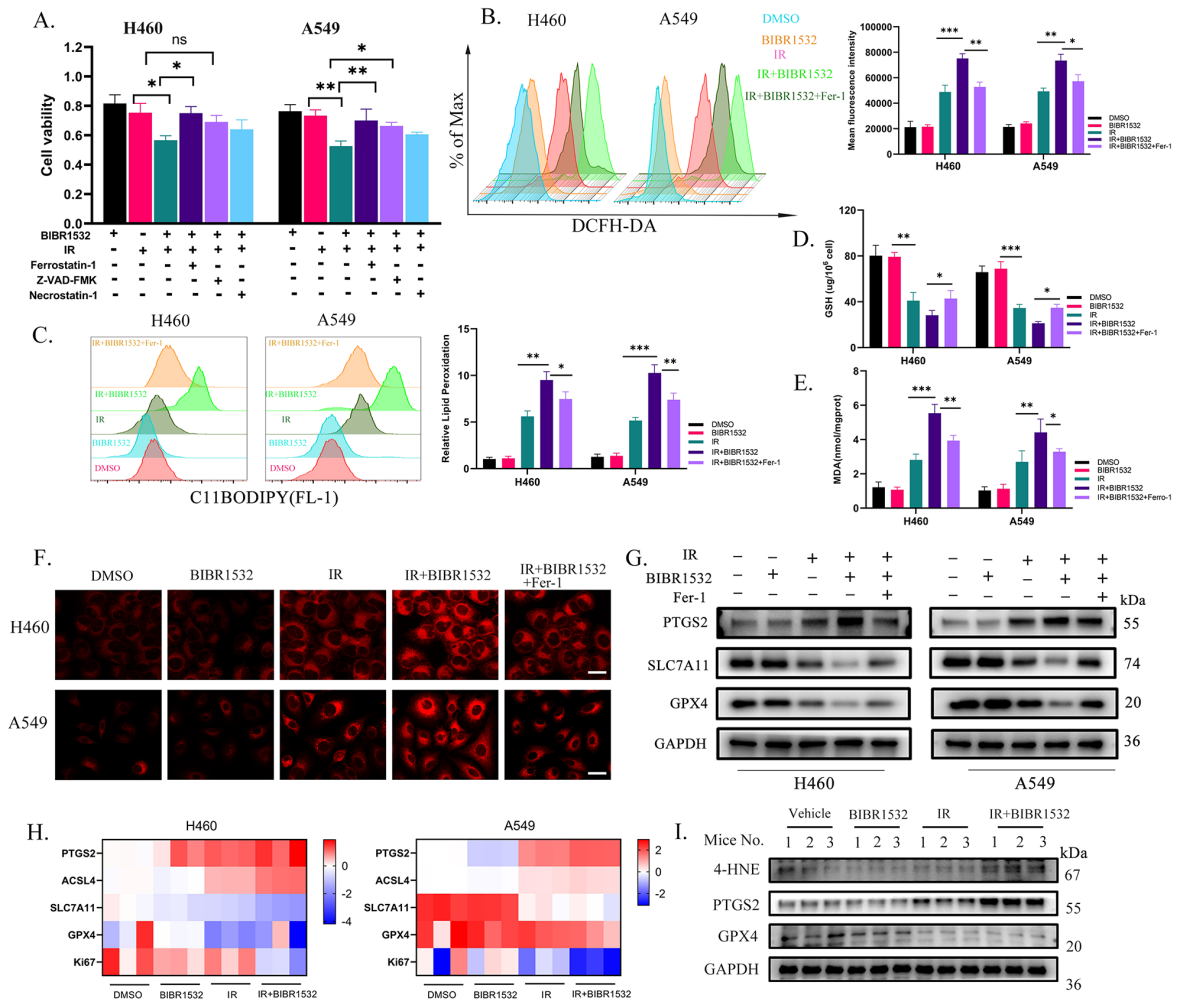
IR combined with BIBR1532 induced the increase of ROS in tumour cells and ferroptosis. **A** Cell viability of H460 and A549 cells to different cell death inhibitors (ferroptosis inhibitors, apoptosis inhibitors, and necrosis inhibitors) after treatment with BIBR1532 and IR was detected by CCK-8 assay. **B, C** DCFH-DA and C11-BODIPY were used to measure intracellular ROS and lipid peroxidation levels after exposure to IR (4 Gy) and BIBR1532 (20 µM for H460, 40 µM for A549) treatment. **D, E** NSCLC cells were pre-treated with or without BIBR1532, and cellular MDA and GSH levels were assessed after 4 Gy irradiation for 24 h. **F** After a 72-hour pretreatment with BIBR1532, cells were irradiated with or without 4 Gy for 48 h, and the intracellular Fe^2+^ concentration was evaluated through image and quantitative analysis. **G** Western blot analysis was conducted to measure the expression levels of PTGS2, SCL7A11, and GPX4 in H460 and A549 cells. **H** qRT-PCR was employed to determine the expression of ferroptosis-related genes in H460 and A549 cells. **I** The Western blot results indicated up-regulation of 4-HNE and PTGS2 expression and decreased GPX4 expression in the tumour tissue of mice treated with BIBR1532 in combination with IR. Each group was replicated three times, and the statistical data is presented as mean ± SD. *P < 0.05, **P < 0.01, ***P < 0.001, ****P < 0.0001

Previous research has demonstrated the induction of ferroptosis in tumour cells by RT, and targeted ferroptosis tumour therapy is currently an area of active investigation [29]. In order to investigate the specific mechanism of cell death induced by BIBR1532 and IR, CCK-8 assay was used to assess the impact of various inhibitors targeting different types of cell death (ferroptosis inhibitor, apoptosis inhibitor and necroptosis inhibitor) on cell viability **(**Fig. 3A**)**. Upon exposure to IR and BIBR1532, it was observed that a selective ferroptosis inhibitor ferrostatin-1 significantly eliminated cell death. However, Z-VAD-FMK (apoptosis inhibitor) and Necrostatin-1 (necroptosis inhibitor) exhibited weaker effects on inhibiting cell death than ferrostatin-1. These findings strongly suggested that ferroptosis played a pivotal role in mediating cell death during treatment with IR alone or in combination with BIBR1532. Ferroptosis, a type of cell death, is typically associated with lipid peroxidation caused by reduced glutathione (GSH) consumption, decreased activity of glutathione peroxidase 4 (GPX4), or increased lipid peroxidation. Ferroptosis is negatively regulated by the solute carrier family 7 A member 11 (SLC7A11), acyl-CoA synthetase long-chain family member 4 (ACSL4), glutathione peroxidase 4 (GPX4) and glutathione (GSH). The levels of total ROS and lipid ROS in H460 and A549 cells were initially quantified **(**Fig. 3B, C**)**, which indicated that the lipid peroxidation was significantly elevated after radiotherapy compared to untreated NSCLC cells, particularly with the administration of BIBR1532. Afterwards, the levels of MDA and GSH were assessed **(**Fig. 3D, E**)**, demonstrating a substantial increase in oxidative product MDA while a notable decrease in antioxidant GSH after treatment with BIBR1532. Ferroptosis induced by radiotherapy led to a significant elevation in intracellular free iron levels. The intracellular Fe2^+^ content in NSCLC cells was measured using the Ferroorange fluorescent probe, the results of which revealed that BIBR1532 significantly increased the intracellular Fe2^+^ level induced by radiotherapy (Fig. 3F and Additional file 2: Fig. S2B). Importantly, the addition of ferroptosis inhibitor ferrostatin-1 during combined treatment of BIBR1532 and radiotherapy effectively inhibited such effect. To further validate that, the expression levels of ferroptosis-related genes were assessed using western blot and q-PCR techniques (Fig. 3G-I and Additional file 2: Fig. S2C-F). The findings revealed that the expressions of PTGS2 and ACSL4 were upregulated, while the expressions of SLC7A11 and GPX4 were downregulated during radiotherapy-induced ferroptosis. These experimental results suggested that BIBR1532 combined with radiotherapy predominantly induced ferroptosis in NSCLC cells.

**Fig. 4:**
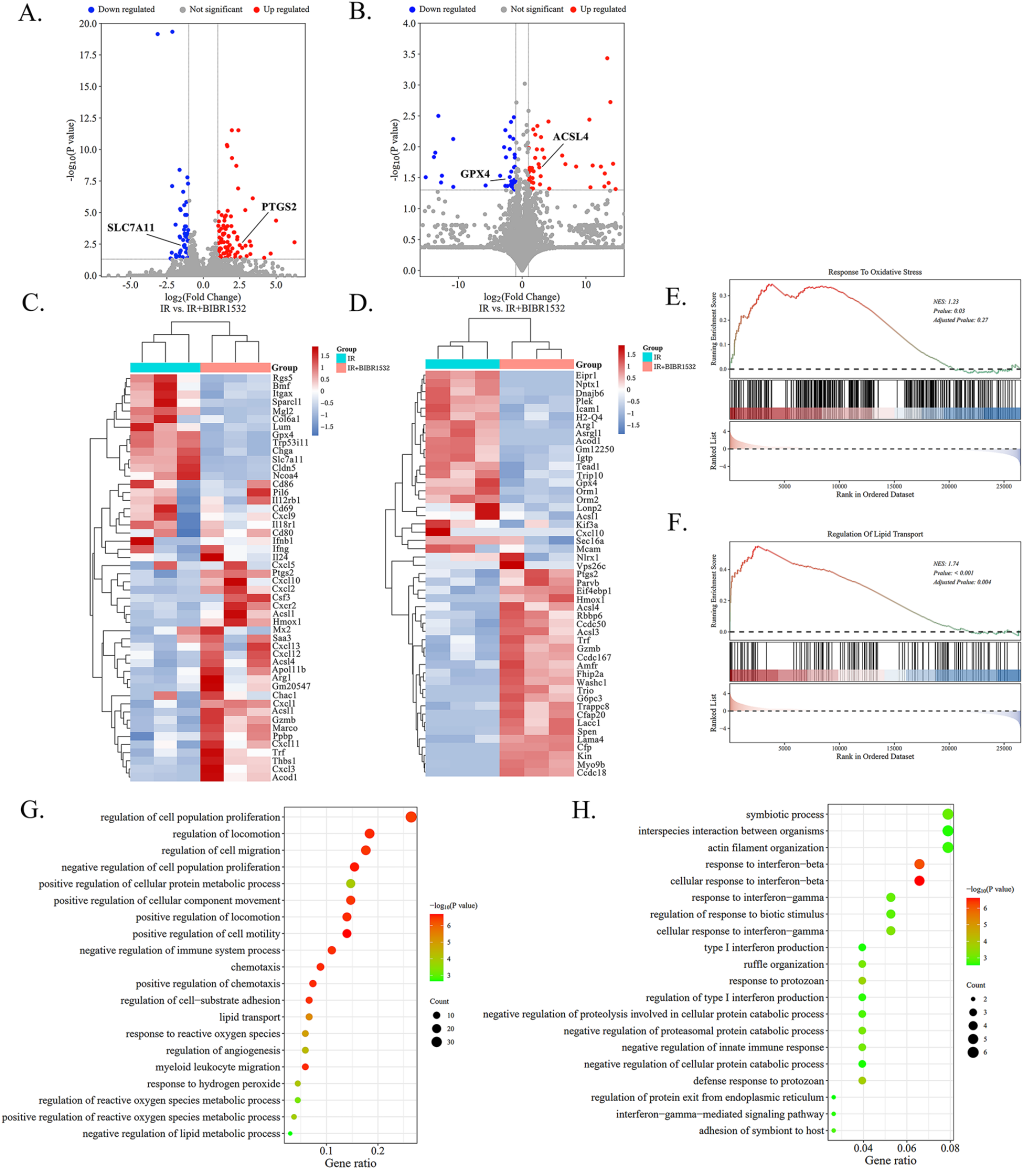
The mechanism of BIBR1532-mediated anti-tumor therapy was evaluated by transcriptome and metabolome analysis. **A, B** The volcano plot shows genes with a fold change of *P* < 0.05. **C, D** Heatmaps depicting significantly up-regulated and down-regulated genes and proteins in tumour tissue. **E, F** GSEA shows genes associated with oxidative stress and lipid transport. **G, H** GO enrichment analysis highlights the primary biological processes in the transcriptome and metabolome

Volcano plot of all significant differentially expressed genes (DEGs). including a total of 85 upregulated DEGs(uDEGs) and 48 downregulated DEGs(dDEGs). Red color means uDEGs, blue color represents dDEGs, and grey color represents genes that are not significantly different in expression. The criterion: |foldchange|>2, *p* < 0.05 is determined as the cutoff value **(**Fig. 4A, B**)**. The results depicted in the volcano map showcase a notable down-regulation in the expression of SLC7A11 and GPX4 following the addition of BIBR1532, juxtaposed with an up-regulation in the expression of PTGS2 and ACSL4. GPX4 is a key player in mitigating phospholipid peroxidation and central to the prevention of ferroptosis. The oxidized form of cysteine, cystine, can also protect against ferroptosis by enhancing GPX4 activity when transported into cells through the systematic cystine-glutamic acid antiporter. Additionally, SLC7A11 plays a crucial role in the cystine/glutamate reverse transport system, shielding cells from lipid peroxidation and ferroptosis. The expression of ACSL4 is a gauge of cellular sensitivity to ferroptosis. These findings align with our previous investigations using Western Blot and qRT-PCR to detect NSCLC cells and tumor tissues, further indicating that the combined treatment of BIBR1532 with RT enhanced the ferroptosis of cells. The heatmap **(**Fig. 4C, D**)** in conjunction with BIBR1532 treatment indicated upregulated expression of the HMOX1 gene and TRF gene. This is in harmony with the Hemin-HMOX1- Fe^2+^ pathway associated with ferroptosis. These outcomes validate the conclusions derived from our prior in vitro experiments, affirming that BIBR1532 in combination with IR can stimulate the ferroptosis of tumour cells. The Gene Set Enrichment Analysis (GSEA) shows genes associated with oxidative stress and lipid transport **(**Fig. 4E, F**)**. The Gene Ontology (GO) analysis revealed that a substantial proportion of differentially regulated pathways was enriched in the modulation of reactive oxygen metabolism, lipid transport, and lipid metabolism **(**Fig. 4G, H**)**.

### IR combined with BIBR1532 enhances mitochondrial damage-induced mtDNA release

**Fig. 5:**
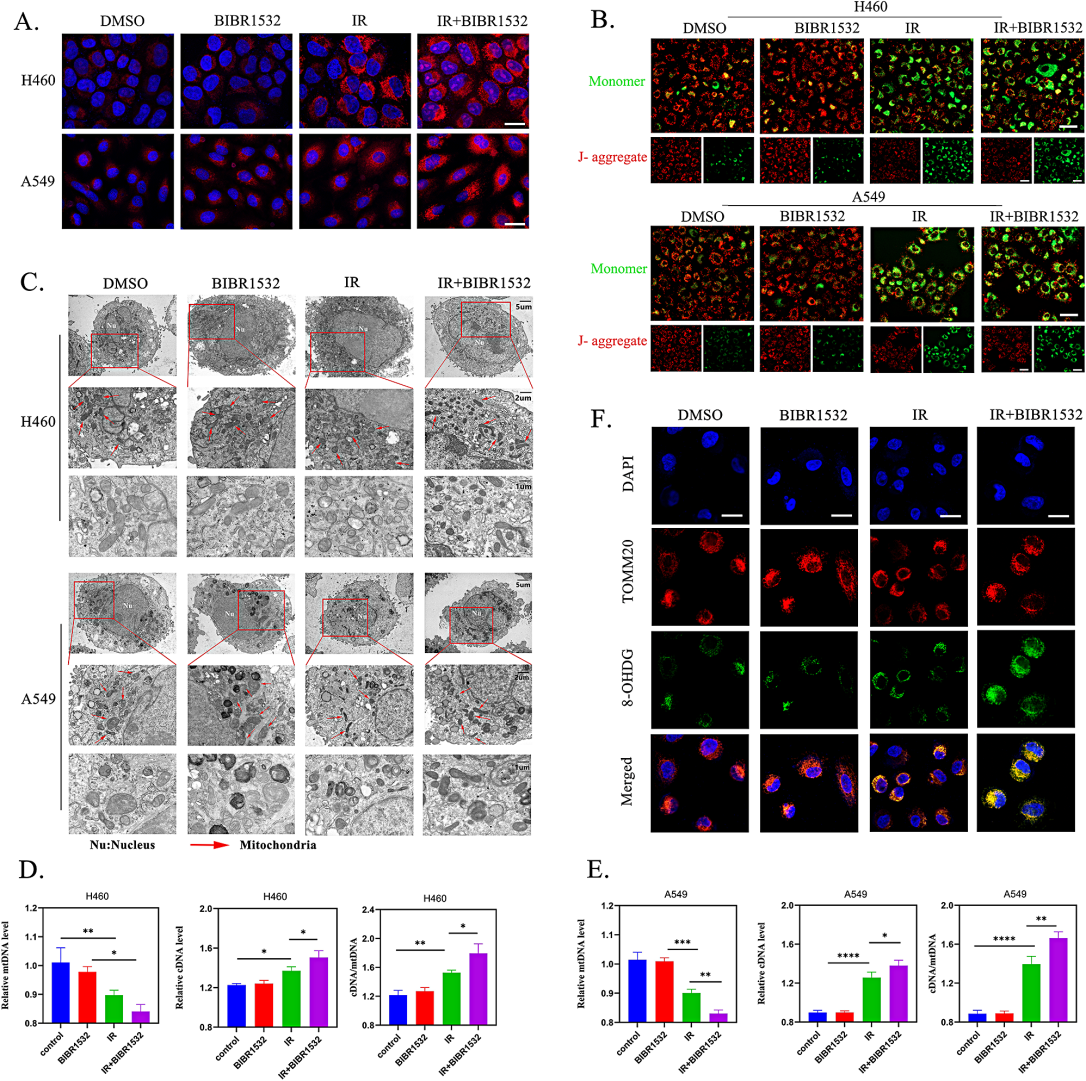
IR combined with BIBR1532 enhanced mitochondrial damage-induced mtDNA release. **A** Representative fluorescence images of mROS in NSCLC cells following different treatments. Scale bar = 20 μm. **B** JC-1 dye was employed to monitor changes in the MMP of NSCLC cells after various treatments. Healthy mitochondria display red fluorescent polymers (J-aggregates), while a reduction in mitochondrial membrane potential results in the green fluorescent monomer form. Scale bar = 50 μm. **C** TEM images of H460 and A549 cells pre-treated with BIBR1532 without irradiation (control) or 24 h after 6 Gy irradiation. (Mitochondria; red arrows, Scale bars: top, 5 μm; middle, 2 μm; below, 1 μm). **D, E** Quantitative analysis of mitochondrial DNA and cDNA in H460 and A549 cells using qRT-PCR. **F** The DNA oxidative damage biomarker 8-OHDG (green) and mitochondrial marker protein TOMM20 (red) were employed to assess mtDNA damage of H460 cells after various treatments. The data is presented as mean ± standard deviation, and each experiment was independently repeated three times. *P < 0.05, **P < 0.01, ***P < 0.001, ****P < 0.0001

Mitochondria play a pivotal role in innate immunity and are particularly susceptible to mitochondrial stress stemming from intracellular ROS. Given the substantial production of intracellular ROS during ferroptosis, it is reasonable to speculate that this process may precipitate mitochondrial oxidative stress. To investigate this hypothesis, we employed the specific mitochondrial ROS indicator, MitoSOX-Red, to gauge the levels of superoxide in the mitochondria of tumour cells following distinct treatments. The results demonstrated a significant amplification of the MitoSOX signal in the group subjected to combined BIBR1532 and RT (Fig. 5A and Additional file 3: Fig. S3A), indicating heightened mitochondrial ROS (mROS) production.

Subsequently, we turned our attention to alterations in mitochondrial membrane potential (MMP) and morphology. The JC-1 staining results revealed that both the RT group and the group receiving RT in conjunction with BIBR1532 exhibited a marked reduction in MMP compared to the control group (Fig. 5B and Additional file 3: Fig. S3B). Furthermore, transmission electron microscopy (TEM) observations exhibited that normal mitochondrial morphology is predominant among the control and BIBR1532 groups. However, the group exposed to IR treatment and the combined treatment group showcased mitochondrial fragmentation and a conspicuous absence of mitochondrial cristae, thus underscoring the considerable mitochondrial damage wrought by ferroptosis **(**Fig. 5C**)**. Subsequently, we sought to ascertain whether mitochondria release mtDNA into the cytoplasm in response to oxidative stress. RT-qPCR results revealed a substantial increase in cytoplasmic dsDNA (cDNA) levels, accompanied by a corresponding decrease in mtDNA levels **(**Fig. 5D, E**)**.

To confirm that oxidative damage primarily targets mtDNA rather than nuclear DNA, we employed immunofluorescence staining with the DNA oxidative damage marker 8-hydroxy-2-deoxyguanosine (8-OHDG) and the mitochondrial marker protein TOMM20 (Fig. 5F and Additional file 3: Fig. S3C-D). The results indicated the pronounced presence of green fluorescence in the IR and IR combined with BIBR1532 groups. Significantly, 8-OHDG, representative of DNA damage, exhibited co-localisation with mitochondrial fluorescence, thus affirming that the majority of oxidative damage is directed towards mtDNA.

These findings provided further confirmation that the mitochondrial oxidative stress induced by ferroptosis, as triggered by RT can stimulate the release of mtDNA into the cytoplasm. Furthermore, the inclusion of BIBR1532 intensifies the ferroptosis induced by RT, thereby leading to a more pronounced release of mtDNA into the cytoplasm.

### BIBR1532 enhanced IR-induced activation of the STING pathway, resulting in anti-tumor immunity

Functioning as a prominent cytoplasmic DNA sensor, cGAS instigates the activation of the STING pathway in both cancer cells and DCs. This activation is succeeded by the phosphorylation of STING, TBK1, and IRF3, leading to the upregulation of IFN-β and the maturation of DCs. These events collectively culminate in the induction of anti-tumor immunity. Having previously established that IR and IR in conjunction with BIBR1532 can induce DNA damage, resulting in the release of cytoplasmic dsDNA, as well as mitochondrial oxidative damage, leading to mtDNA release into the cytoplasm, we hypothesized that BIBR1532 might have implications for the immune microenvironment.

**Fig. 6:**
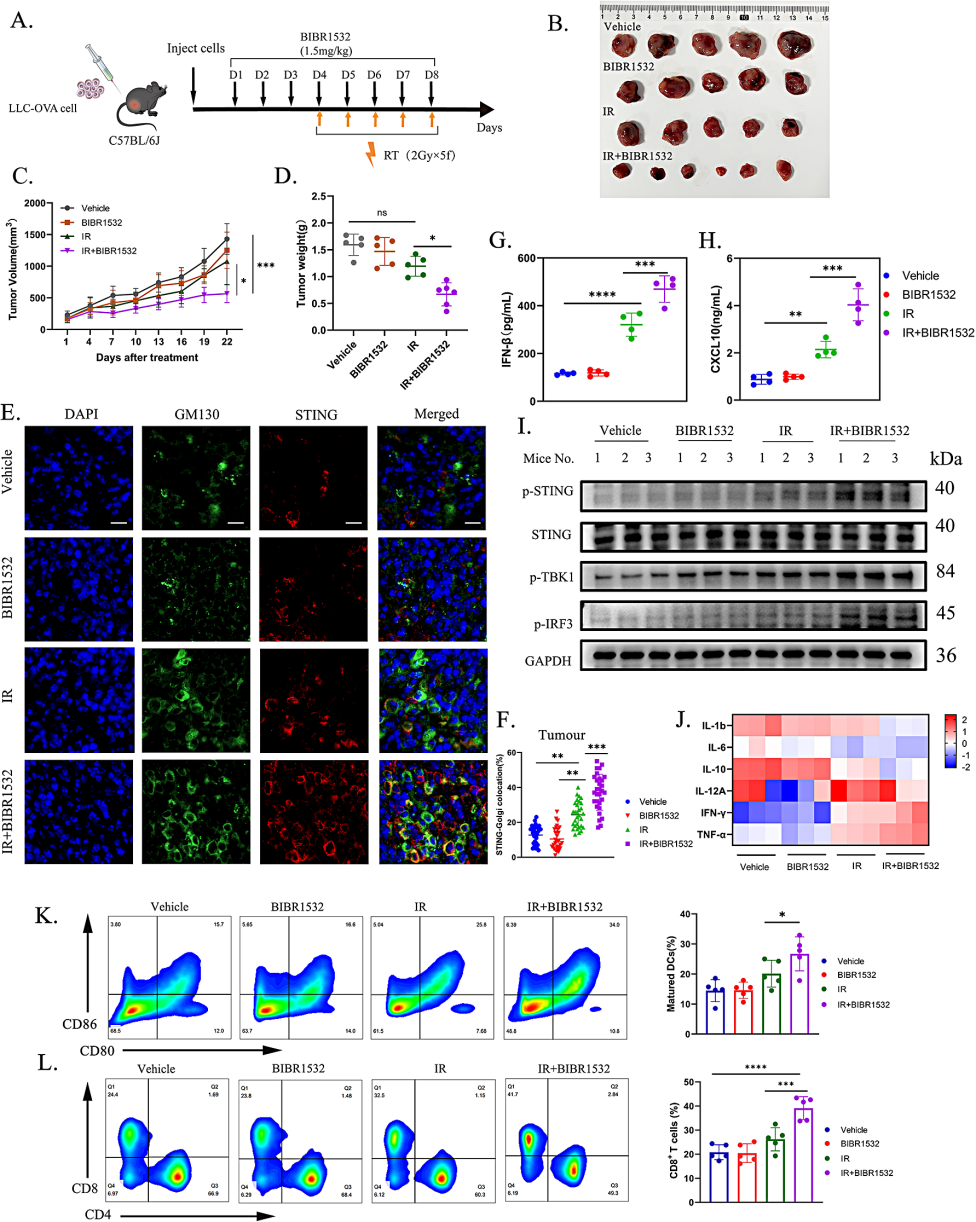
BIBR1532 combined with IR increased STING pathway activation-mediated anti-tumor immunity. **A** Schematic representation of the treatment process for LLC-OVA tumor-bearing mice. Mice were randomly assigned to groups when tumour volume reached 100 mm³ and treatment was initiated. The tumour volume and body weight of the mice were recorded every three days. **B-D** The figure displays the tumour volume, tumour weight, and growth curve of mice in different treatment groups. **E** Representative images depicting the localization of STING (red) and GM130 (cis-Golgi, green) of tumour tissue. Scale bar = 50 μm. **F** The co-localization of STING and GM130 signals as a proportion of total STING signals, the COLOC analysis by selecting 30 cells from each group for analysis. **G, H** Concentrations of IFN-β and CXCL10 in the tumour were determined using ELISA. Each group was replicated three times, and the statistical data is presented as mean ± SD. **I** Western blot results show that IR combined with BIBR1532 increased the expression of phosphorylated STING, TBK1, and IRF3. **J** The expression of inflammatory factors and cell effector factors in tumour tissue was assessed by qRT-PCR. **K, L** Flow cytometry was used to detect the proportion of mature DCs and CD8 ^+^ T cytotoxic lymphocytes in tumour tissue. Statistical data is expressed as mean ± SD. *P < 0.05, **P < 0.01, ***P < 0.001, ****P < 0.0001, ns = not significant

In an initial experiment, LLC-OVA cells were subcutaneously inoculated into immunocompetent C57BL/6J mice and subsequently treated **(**Fig. 6A**)**. The results illustrated that the combined treatment of BIBR1532 with IR significantly impeded tumour growth **(**Fig. 6B-D**)**. A key marker of STING activation involves the translocation from the endoplasmic reticulum to the Golgi apparatus. In the group subjected to RT combined with BIBR1532, heightened co-localization of STING and the cis-Golgi marker protein GM130 was observed **(**Fig. 6E, F**)**, signifying the optimised activation of the STING/type I IFN pathway in the context of combination therapy.

Subsequently, we used enzyme-linked immunosorbent assay to detect the expression level of IFN-β and CXCL10 (Fig. 6G, H ), Western blotting to detect the level of STING, TBK1, IRF3 Fig. 6I and Additional file 4: Fig.S4**A)**. Alongside the alteration in expression of tumour-related inflammatory factors and cellular effectors following diverse treatments **(**Fig. 6J**)**. The findings corroborated the notion that combination therapy leads to the most extensive activation of the STING pathway, fostering the maturation of DCs and augmenting the infiltration of CD8 ^+^ T cells **(**Fig. 6K, L**)**. In addition, after BIBR1532 combined with radiotherapy, the central memory T cells (CD44^+^CD62L^+^CD8^+^T) showed the most significant increase, while depleted T cells (TIM-3^+^PD-1^+^CD8^+^T) and Treg cells (CD25^+^Foxp3^+^ CD4^+^T) showed a significant decrease. These results indicate that BIBR1532 combined therapy has the most significant effect on immune activation (Additional file 4: Fig. S4 B-E).

### Enhanced systemic anti-tumour efficacy with the combination of BIBR1532, RT, and immune checkpoint inhibitors

Immunotherapy has substantially improved the prognosis of patients with advanced NSCLC. Targeting the immunosuppressive pathway involving the programmed death receptor-1 (PD-1) and its ligand (PD-L1) through immune checkpoint inhibition (ICI) therapy can further enhance the effectiveness of ICI in NSCLC patients who received RT [30]. The global multicenter phase III clinical trial, known as the PACIFIC study, demonstrated significant enhancements in progression-free survival and overall survival rates for patients who received the PD-L1 monoclonal antibody, Durvalumab, following concurrent chemoradiotherapy [31]. Nonetheless, a considerable portion of patients still fail to experience the long-term survival benefits they need. Therefore, the search for synergistic agents capable of maximising the combination of RT and immunotherapy is imperative.

**Fig. 7:**
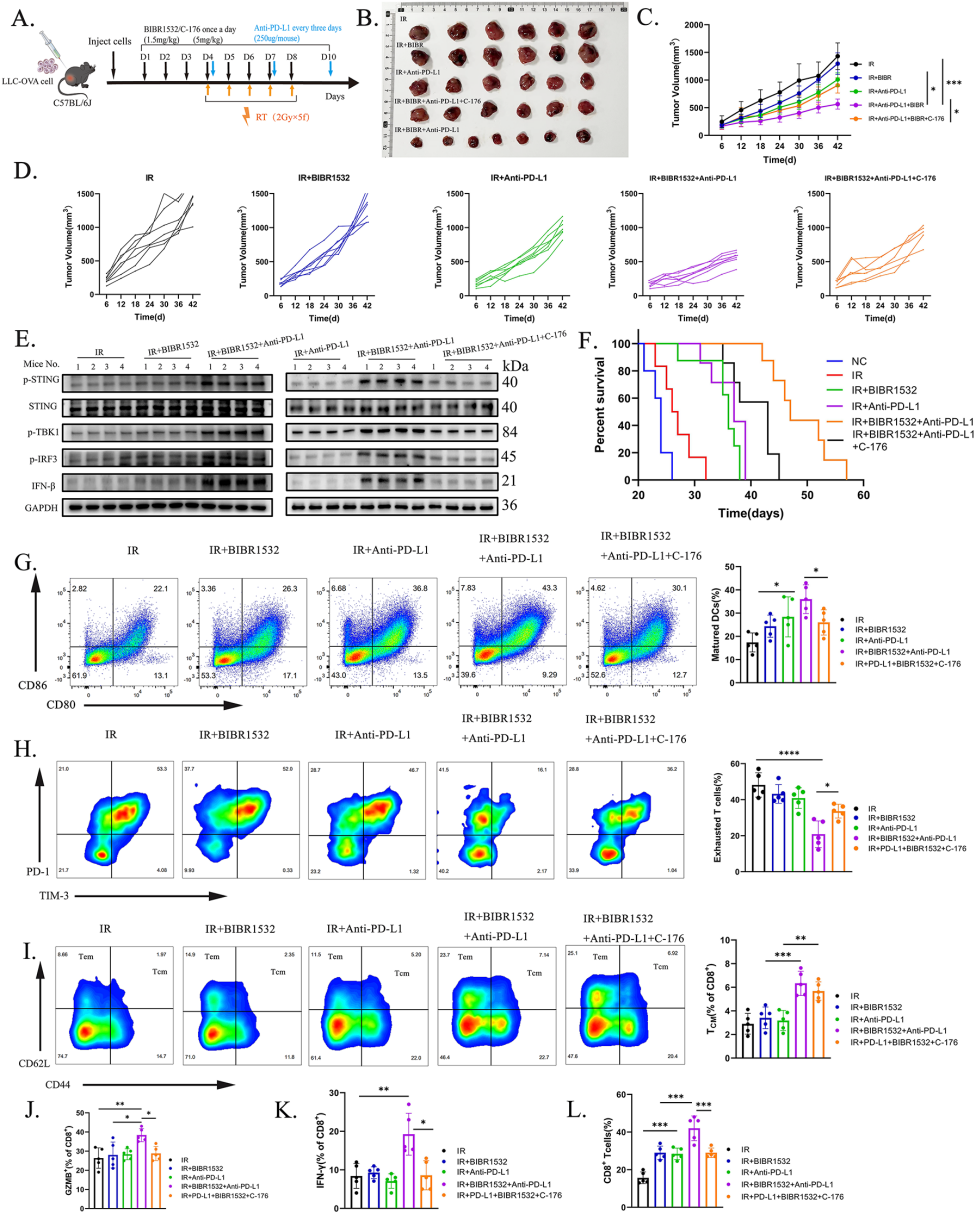
BIBIR1532 combined with RT and immune checkpoint inhibitors enhances systemic anti-tumor efficacy. **A** Schematic diagram outlining the treatment process for LLC-OVA tumor-bearing mice. Mice were randomly divided into groups when tumour volume reached 100 mm³ and treatment commenced. Tumor volume and body weight were measured every three days. **B-D** The figure displays the tumour volume of mice after different treatments, along with the growth curve of transplanted tumours and individual tumour growth curves. **E** Several proteins of the cGAS/STING signalling pathway were detected in subcutaneous tumours of LLC-OVA tumour-bearing mice. **F** Survival analysis of mice following various treatments. **G** Representative flow cytometry and quantification of mature DCs. **H** Quantification of exhausted T cells in tumour tissue after different treatments. **I** Representative flow chart and quantification of TCM in the spleen. **J-L** The percentage of GZMB in peripheral blood and IFN-γ in tumour tissue, along with the percentage of CD8 ^+^ T cells in tumour tissue. Statistical data are expressed as mean ± SD.*P < 0.05, **P < 0.01, ***P < 0.001, ****P < 0.0001

We have ascertained that BIBR1532, when combined with RT, can enhance the tumour immune microenvironment (TIME) by activating the STING pathway. However, it is unclear whether the application of ICI for immunotherapy can further enhance the anti-tumor immune activation effect induced by BIBR1532 combined with radiation therapy. To address this, we subjected LLC-OVA tumour-bearing mice to various therapeutic regimens **(**Fig. 7A**)**. The results exhibited that the triple therapy group, consisting of BIBR1532 in combination with IR and Anti-PD-L1, displayed the most pronounced inhibitory effect on tumour growth when compared to the IR combined with Anti-PD-L1 group and the IR combined with BIBR1532 group. It is noteworthy that the treatment group receiving the STING inhibitor (C-176) mitigated the anti-tumour effect of the triple therapy **(**Fig. 7B-D**)**.

Subsequently, we investigated the effects of different therapeutic approaches on the cGAS/STING pathway. The results unveiled that the triple therapy increased the phosphorylation of STING, TBK1, IRF3, and IFN-β in comparison to other treatment groups, thus confirming its activation of the cGAS/STING pathway (Fig. 7E and Additional file 5: Fig. S5 A). To investigate whether BIBR1532 combined with RT could improve prognosis in vivo, the survival analysis results **(**Fig. 7F**)** showed that the combination of BIBR1532 with RT did not prolong survival time compared with RT alone, but the prognosis was significantly improved with the addition of anti-PD-L1 treatment (median survival time, 36 days IR + BIBR1532 vs. 47 days IR + BIBR1532 + Anti-PD-L1, *p* < 0.0001; 47 days IR + BIBR1532 + Anti-PD-L1 vs. 43 days IR + BIBR1532 + Anti-PD-L1 + C-176, *p* = 0.006. The peripheral blood and vital organs of mice were assessed post-euthanasia to evaluate the toxic effects of different treatments. The findings revealed no evidence of myelosuppression or acute organ injury in each treatment group. (Additional file 5: Fig. S5 B, C). We then delved further into the ramifications of distinct therapies on TIME. In line with our predictions, the triple therapy maximally activated mature DCs, thereby enhancing the cytotoxicity of CD8 ^+^ T cells **(**Fig. 7G, L**)**. Moreover, in contrast to other groups, the triple group reduced the proportion of depleted T cells (CD8 ^+^ T PD-1^+^ TIM-3^+^) in tumour tissue **(**Fig. 7H**)**. Concurrently, with regards to immune memory, the triple group raised the percentage of central memory T (Tcm) cells in the spleen **(**Fig. 7I**)**. Additionally, the triple therapy elevated the levels of GZMB in peripheral blood and IFN-γ in tumour tissue **(**Fig. 7J, K**)**. The number of tumor-infiltrating Treg cells can be significantly inhibited after combined treatment (Additional file 5: Fig. S5 D). Notably, the addition of C-176 partially reversed the anti-tumour immune effect of triple therapy.

**Fig. 8:**
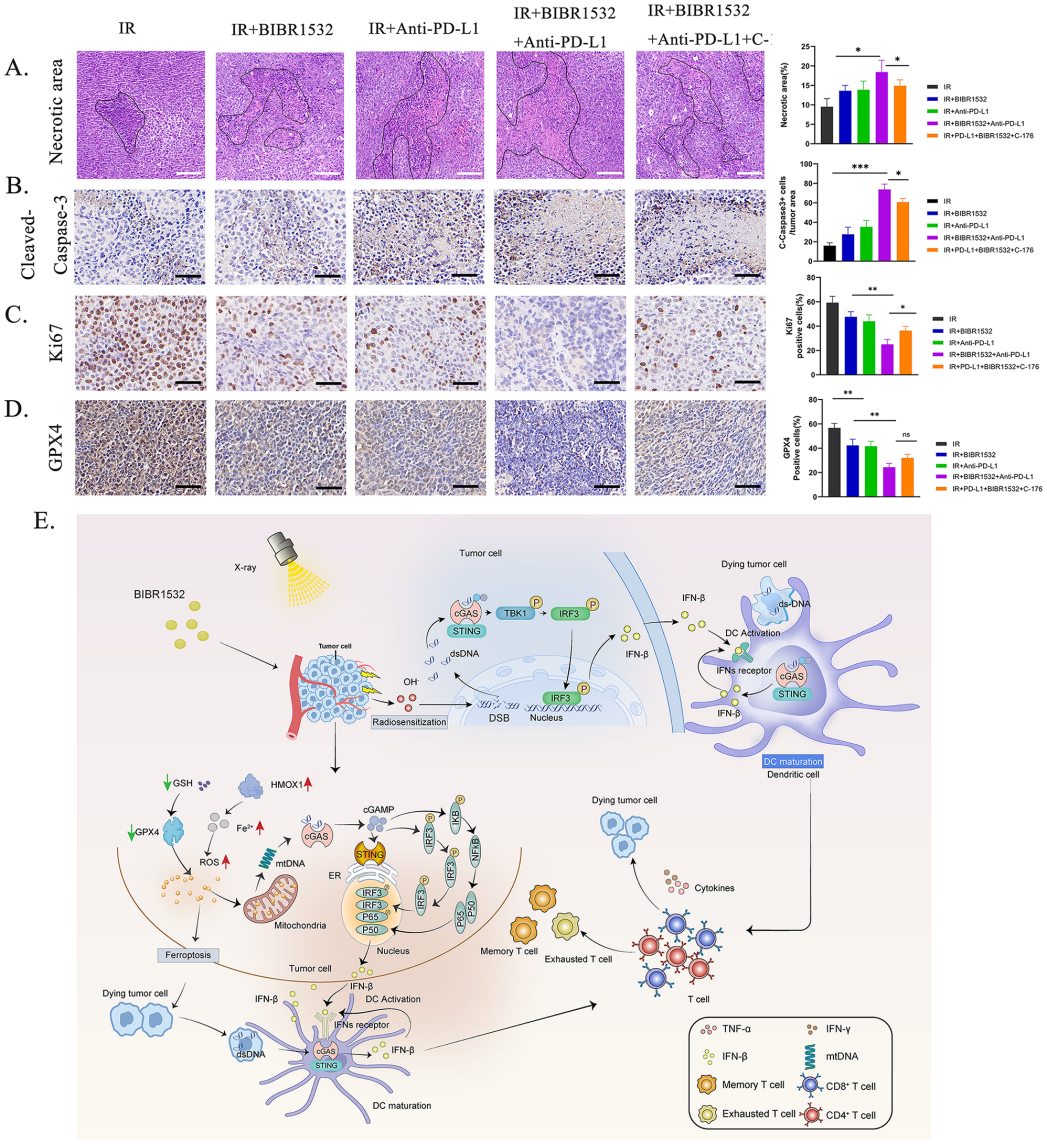
Schematic of the activation mechanism of BIBR1532 combined with RT-mediated cGAS-STING pathway. **A** The area of tumour necrosis in different treatment groups. **B** Expression levels of cleaved-caspase3 in different treatment groups were assessed through IHC analysis. **C** The ratio of Ki-67 positive cells in the five groups of mice. **D** Expression levels of GPX4 in different treatment groups were assessed through IHC analysis. **E** The mechanism of BIBR1532 combined with RT-mediated cGAS-STING pathway activation to initiate anti-tumour immunity and enhance ICI for inhibiting local tumours

The tumour tissue was collected on day 42 and subsequently underwent histological examination using HE staining as well as immunohistochemical analysis. **(**Fig. 8A-D). The findings reaffirmed that higher extent of necrotic areas in the triple group compared to the other groups. Furthermore, the proliferation of tumour cells was significantly lower in the triple group than in other treatment groups. For a more insightful observation of potential ferroptosis effects, GPX4 staining of tissue sections revealed that the triple group exhibited the lowest GPX4 expression. The addition of C-176 did partially reverse this effect, although the difference was not statistically significant. In summary, triple therapy offers the most substantial enhancement in the efficacy of radioimmunotherapy.

## Discussion

IR induces telomere dysfunction, resulting in the fusion of chromosome ends, ultimately leading to a catastrophic failure in cell mitosis. And this is a fundamental mechanism underlying the demise of solid tumor cells due to radiation exposure [32]. BIBR1532 emerges as a promising hTERT-specific active site inhibitor, capable of impairing DNA repair through the induction of telomere dysfunction. It effectively halts the growth of tumour cells both in vitro and in vivo thus enhances their susceptibility to ionizing radiation [33, 34]. In our current investigation, we have observed that BIBR1532 significantly impedes the activation of the ATM/ATR-mediated DNA damage repair pathway subsequent to radiotherapy. This inhibition effect curbs the proliferation of tumour cells and restrains the growth of tumour volume. Furthermore, we have confirmed its radiosensitizing effect in the RT of NSCLC, both in vitro and in vivo. It’s noteworthy that in malignant solid tumors and hematological tumor cells the telomerase exhibits an increased activity under low-dose radiation conditions, but as the radiation doses rise, telomerase activity returns to baseline levels or even below baseline [35, 36]. Consequently, the radiosensitizing influence of BIBR1532 on hypofractionated radiotherapy is not as conspicuous as that observed in conventional fractionated radiotherapy. BIBR1532 operates by curtailing telomerase activity and is correlated with the initial telomere length, thereby limiting its utility over extended durations and at higher doses [37, 38]. Our research has revealed that the combination of IR with a low concentration of BIBR1532 effectively suppresses the growth of tumour cells, meanwhile, we have not observed any significant bone marrow suppression or acute organ damage in our in vivo experiments. This finding attests to the effectiveness and safety of the treatment strategy which combined IR with a non-toxic dose level of BIBR1532 in locally advanced NSCLC.

More significantly, our study delves into the impact and potential mechanism of low-dose BIBR1532 in conjunction with radiotherapy on the TIME. The co-administration of BIBR1532 in RT activates the cGAS/STING signalling pathway, although BIBR1532 alone does not elicit this response. This activation of cGAS in synergy with IR augments its sensitivity to cytoplasmic dsDNA, possibly attributable to the effect of BIBR1532, which could affect telomerase activity alone. It’s important to note that before the telomeres are shortened to a critical length, there is typically a substantial time lag [39]. Nevertheless, ionizing radiation (IR) induces exogenous DNA damage, directly causing breaks in chromosomal DNA and generating substantial DSBs. At this juncture, the non-homologous end joining (NHEJ) pathway initiates an expeditious effort to repair these DSBs. However, this template-free repair mechanism may inadvertently give rise to structurally aberrant chromosomal fragments. Subsequently, these atypical fragments become improperly separated and culminated with the formation of micronuclei [40, 41]. Micronuclei could rupture the dsDNA and release it into the cytoplasm, thereby activating the cytoplasmic sensor cGAS. Cognizant of this activation, cGAS catalyses the synthesis of cyclic GMP-AMP (cGAMP), in turn triggering the innate immune response [42]. Notably, BIBR1532 intensifies the damage inflicted by IR on DNA, thereby enhancing the phosphorylation of STING, TBK1, and IRF3 proteins in both tumour cells and dendritic cells (DCs). This elevation in phosphorylation levels leads to an increased expression of interferon-beta (IFN-β), fostering the maturation of DCs and inciting an anti-tumour immune response.

Our investigation also revealed an elevation in intracellular Fe^2+^ content and an increase in HMOX1 expression upon the addition of BIBR1532 to RT. Furthermore, the synthesis of GSH decreased, and the expression of GPX4 declined in the combined treatment group. These findings suggest that the combined therapy may elicit ferroptosis through the synergistic impact of both the classical ferroptosis pathway (System Xc-GSH-GPX4 pathway) and the non-classical ferroptosis pathway (Hemin-HMOX1- Fe^2+^ pathway) [43, 44].

A decrease in MMP was observed in both the RT group and the RT combined with BIBR1532 group. Furthermore, the TEM revealed the disappearance of mitochondrial debris and mitochondrial cristae, alongside an increase in lipid droplets and autophagosomes, signifying extensive mitochondrial damage. While nuclear DNA is typically rigorously confined within the nucleus, guarded by densely packed chromatin, mtDNA follows a distinct set of rules. mtDNA functions as a crucial organelle in innate immunity and is notably sensitive to ROS [45–47].

To evaluate the extent of mtDNA damage, we employed MitoSOX-Red, a mitochondria-specific reactive oxygen species indicator, to gauge superoxide levels in tumour cells following various treatments. Moreover, co-localization of the specific DNA oxidative damage marker, 8-hydroxy-2-deoxyguanosine (8-OHDG), with the mitochondrial protein TOMM20 confirmed that mtDNA had indeed been compromised by mROS and oxidative stress. Subsequently, our analysis revealed an increase in cDNA content coupled with a decrease in mtDNA content in the cytoplasm, indicating the release of mtDNA into the cytoplasm.

The cytoplasmic DNA sensor, cGAS, plays a pivotal role in recognising and converting CTP and ATP into a second messenger called cGAMP. The adaptor protein STING forms a dimer upon binding to cGAMP, leading to the recruitment of TBK1, which triggers the phosphorylation of IRF3 and its subsequent nuclear translocation. The activation of STING is characterised by its translocation from the endoplasmic reticulum to the Golgi apparatus [48]. Thus, in the RT combined with BIBR1532 group, we observed a heightened co-localization of STING with the cis-Golgi marker protein GM130. This points to the activation of the STING/type I IFNs pathway, which, in turn, induces cross-activation of DCs and the activation of tumour-specific CD8 ^+^ T cells.

The TIME constitutes a dynamic network governed by intercellular communication, and plays a pivotal role in cancer progression and metastasis [49]. IR has the capacity to activate the innate immune system, rendering tumour antigen-presenting cells and T cells more receptive. It does so by inducing immunogenic cell death and the release of neoantigens, thereby augmenting the diversity of T cell antigen recognition. Essentially, this transforms the tumour into an in-situ vaccine [50].

Nevertheless, certain studies have pointed out that IR can stimulate the DNA damage response pathway, leading to an increase in PD-L1 expression and the formation of an inhibitory tumour immune microenvironment [51]. It is important to note that the effect of IR on the TIME varies with the radiation dosage. High-dose radiation primarily heightens antigen release and presentation through immunogenic cell death, while low-dose radiation can induce immune regulation. Lymphocytes, for instance, are notably sensitive to radiation, and low-dose irradiation can both expedite the apoptosis of most naive lymphocytes and contribute to the activation of effector T cells [52, 53]. Moreover, low-dose radiation can facilitate the release of inflammatory cytokines and damage-associated molecular patterns, thereby enhancing the mobilisation and activity of immune cells. Research has shown that when low-dose irradiation is combined with ICIs, it can synergistically trigger both natural and adaptive immunity, leading to the transformation of the TIME from a pro-tumorigenic or immunosuppressive state to one characterised by the infiltration of cytotoxic T cells that actively target and destroy cancer cells. This approach has shown a remarkable increase in the complete response rate in preclinical tumour models [54, 55].

In the context of this study, the triple therapy involving BIBR1532 in conjunction with IR and Anti-PD-L1 substantially bolstered the activation of mature DCs. This treatment fostered the proliferation and immunological memory of cytotoxic CD8^+ T^ cells while mitigating the depletion of these cells. Consequently, the triple therapy demonstrated superior improvements in the TIME compared to other treatment regimens. Notably, this positive trend was attenuated upon the addition of STING inhibitors, underscoring the fact that this combination therapy induces anti-tumour immune effects by activating the cGAS/STING signalling pathway. This leads to DCs maturation, enhances the proliferation and immunological memory of CD8 ^+^ T cells.

In this study, our investigation explored the impact and potential mechanism of BIBR1532 on the TIME following RT. However, it’s imperative to acknowledge that mitochondria also house DNA with immunogenic potential. While classical nuclear DNA damage repair proteins can influence the immunogenicity of mtDNA [56], we have limited knowledge about the manner in which mtDNA damage repair and how mtDNA comes to be located in the cytoplasm. Furthermore, primary tumours and distant tumours often display heterogeneity in the TIME [57], implying that they may respond differently to the same treatment approach. Consequently, the effect of the treatment model employed in this study on distant tumours remains unclear, warranting further investigation in future studies.

In summation, our study has elucidated the regulatory impact of the highly selective telomerase inhibitor BIBR1532 when combined with RT on the radiosensitivity and radioimmunotherapy of NSCLC **(**Fig. 8E**)**. BIBR1532 notably augments the capacity of RT to generate dsDNA and facilitates the release of mtDNA induced by ferroptosis-related mitochondrial stress. Both of these phenomena set in motion the cGAS-STING pathway, rendering tumours more amenable to RT and radioimmunotherapy.

### Electronic supplementary material

Below is the link to the electronic supplementary material.

Supplementary Material 1

Supplementary Material 2

Supplementary Material 3

Supplementary Material 4

Supplementary Material 5

Supplementary Material 6

Supplementary Material 7

Supplementary Material 8

## Data Availability

All data that support the findings of this study were available from the authors upon reasonable request.

## Abbreviations

ATM: Ataxia Telangiectasia Mutant Kinase
ATR: Ataxia Telangiectasia and Rad3-Related Protein
ACSL: 4-Acyl-Coa Synthetase Long-Chain Family Member 4
cGAS: Cyclic Guanosine Monophosphate (Gmp)-Adenosine Monophosphate (Amp) Synthase
CHK1: Checkpoint Kinase1
CHK2: Checkpoint Kinase2
CXCL10: CXC-Chemokine Ligand-10
DSBs: DNA Double-Strand Breaks
dsDNA: Double-Stranded DNA
DC: Dendritic Cell
DDR: DNA Damage Response
DNA-PKcs: DNA Dependent Protein Kinase Catalytic Subunit
FINs: Ferroptosis Inducers
GPX4: Glutathione Peroxidase 4
GSH: Glutathione
HMOX1: Heme Oxygenase-1
hTERT: Human Ribonucleoprotein Reverse Transcriptase
4-HNE: 4-Hydroxynonenal
IR: Ionizing Radiation
IFN: Type I Interferon
ICI: Immune Checkpoint Inhibition
IRF3: Interferon Regulatory Factor 3
LDH: lactate Dehydrogenase
mROS: Mitochondrial ROS
mtDNA: Mitochondrial DNA
MDA: Malondialdehyde
MMP: membrane potential
8-OHDG: 8-Hydroxy-2-Deoxyguanosine
PD-1: Programmed Death Receptor-1
PD-L1: Programmed Death Receptor Ligand-1
PTGS2: Cyclooxygenase-2
RT: Radiotherapy
ROS: Reactive Oxygen Species
STING: Stimulator of Interferon Genes
SLC7A11: Cystine/ Glutamate Transporter
TBK1: Tank-Binding Kinase 1
TOMM20: Translocase of Outer Mitochondrial Membrane 20 Homolog
TIME: Tumour Immune Microenvironment
