# Supplementary figures and images for: BIBR1532 combined with radiotherapy induces ferroptosis in NSCLC cells and activates cGAS-STING pathway to promote anti-tumor immunity
Source: J Transl Med. 2024 May 30;22:519. doi: 10.1186/s12967-024-05331-3 (PMC11138045; doi:10.1186/s12967-024-05331-3)

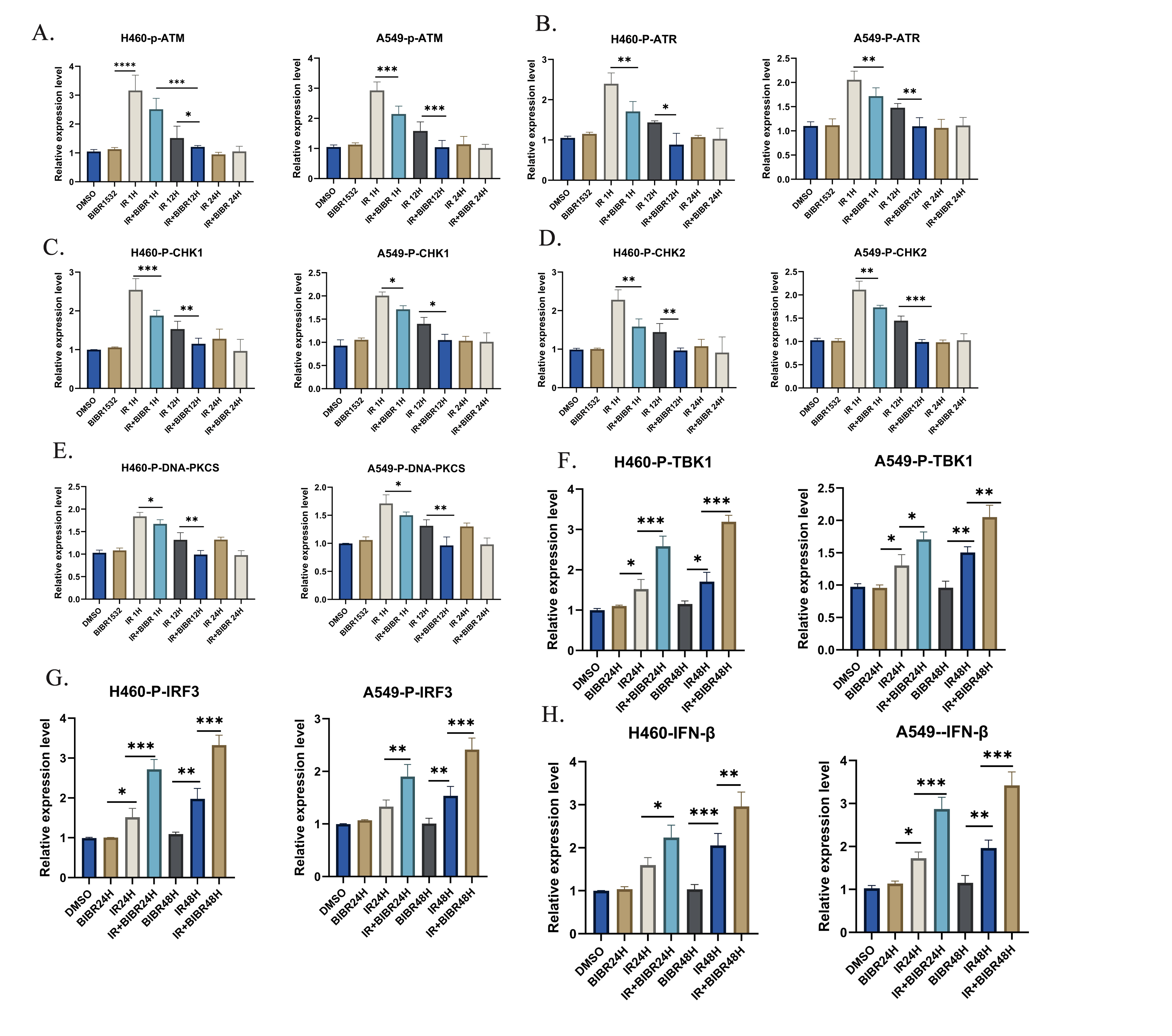

Supplement: Supplementary file 1 — Supplementary Material 1 [file 12967_2024_5331_MOESM1_ESM.tif]

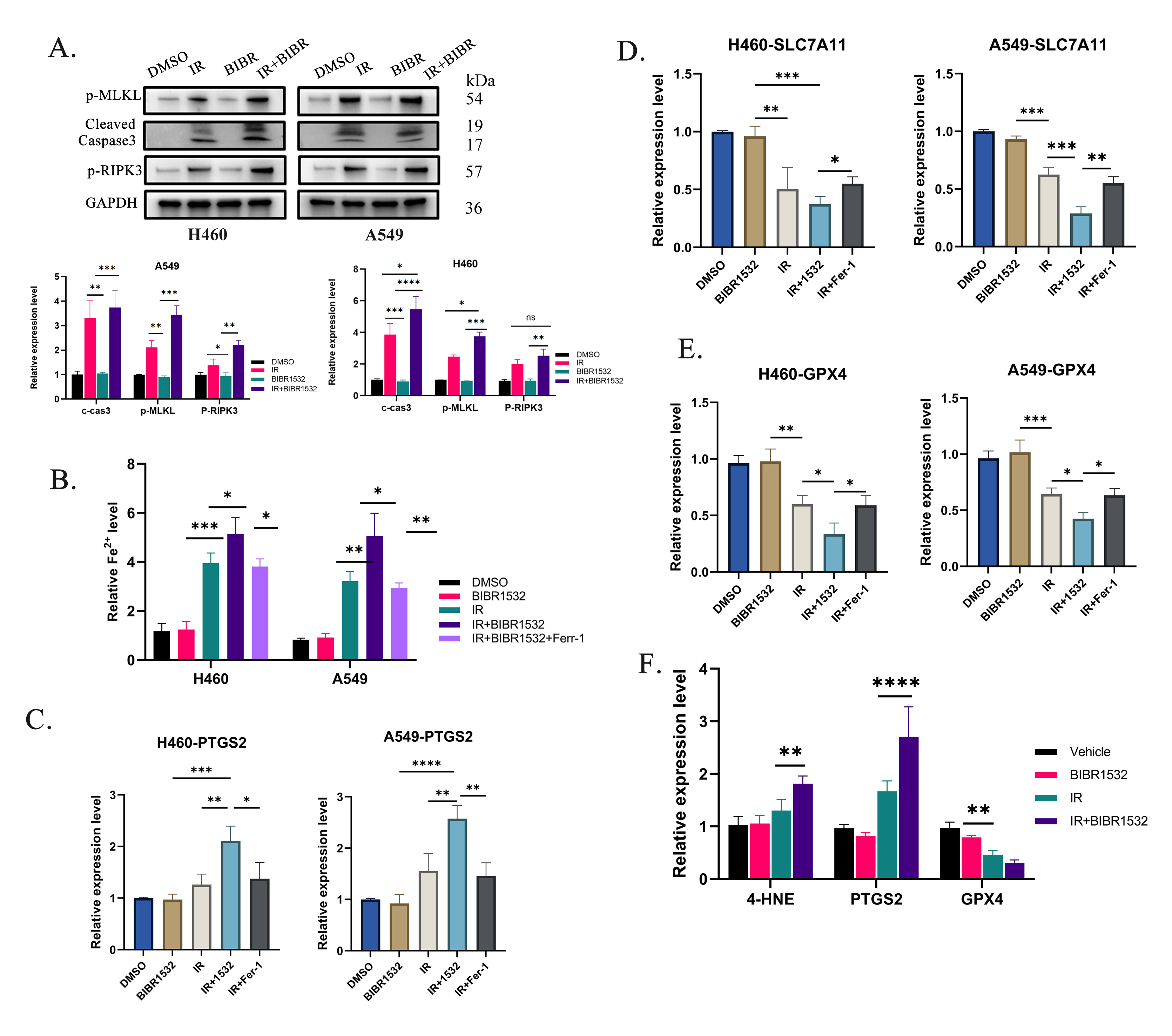

Supplement: Supplementary file 2 — Supplementary Material 2 [file 12967_2024_5331_MOESM2_ESM.tif]

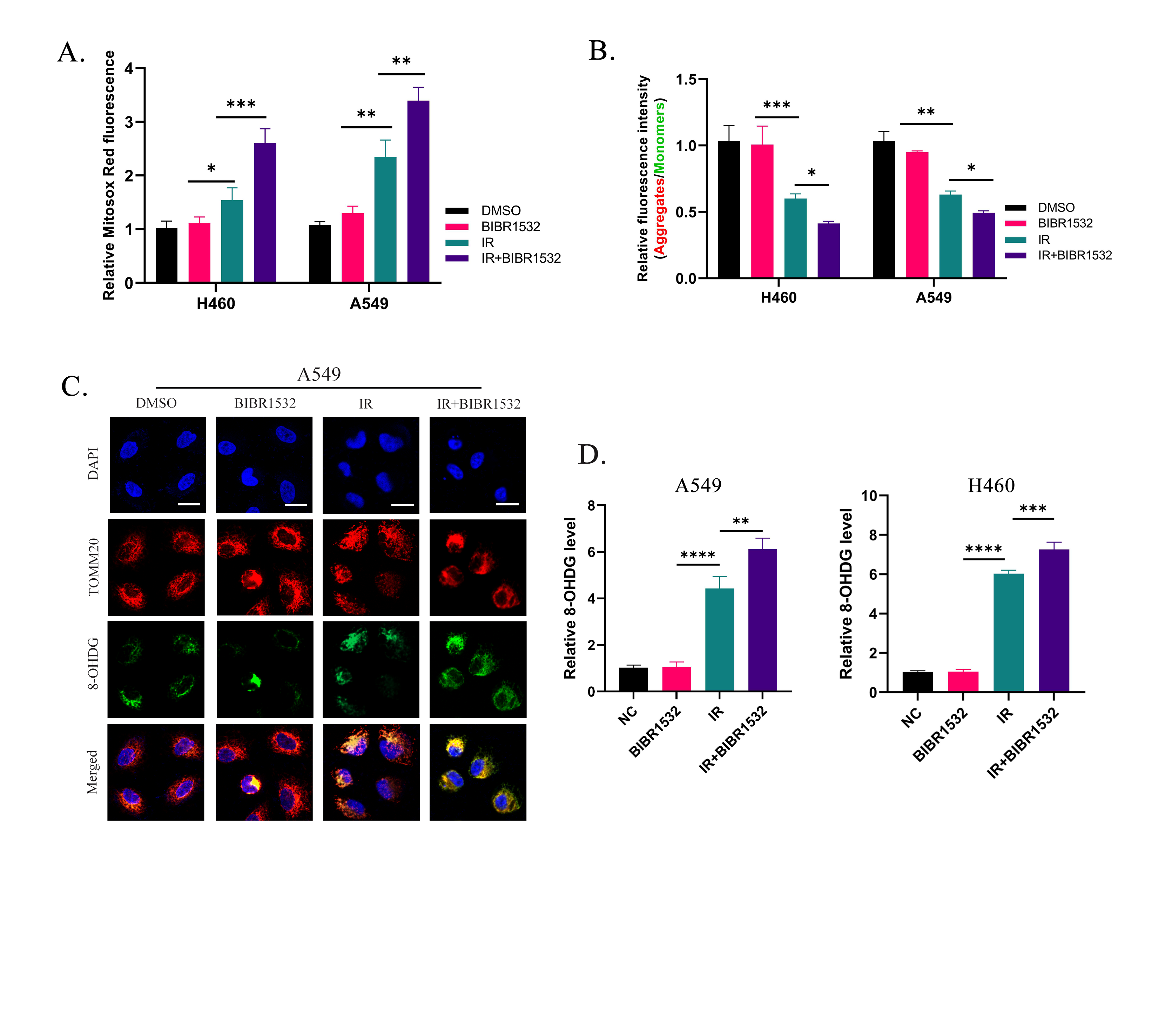

Supplement: Supplementary file 3 — Supplementary Material 3 [file 12967_2024_5331_MOESM3_ESM.tif]

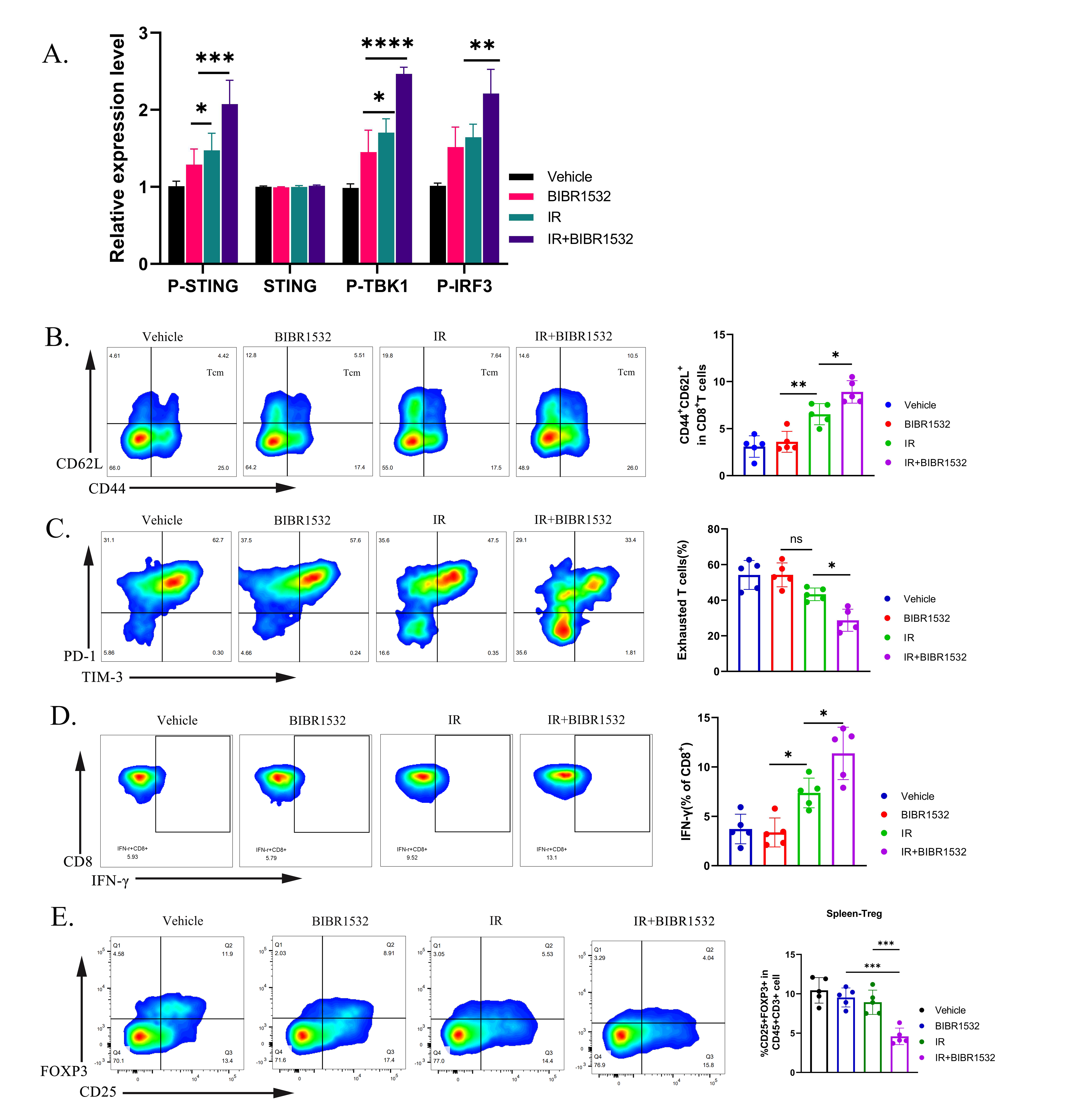

Supplement: Supplementary file 4 — Supplementary Material 4 [file 12967_2024_5331_MOESM4_ESM.tif]

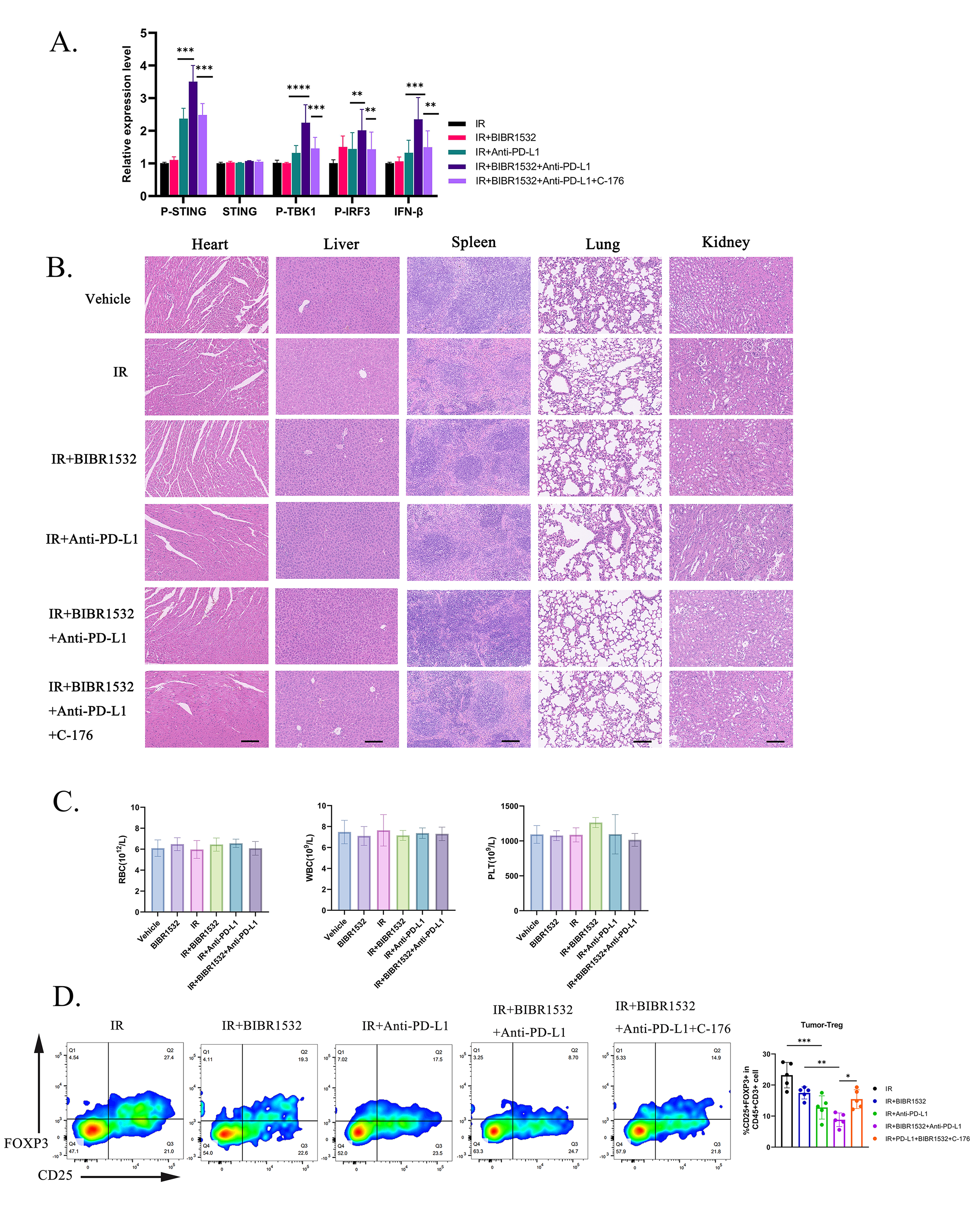

Supplement: Supplementary file 5 — Supplementary Material 5 [file 12967_2024_5331_MOESM5_ESM.tif]
