# Supplementary material for: BIBR1532 combined with radiotherapy induces ferroptosis in NSCLC cells and activates cGAS-STING pathway to promote anti-tumor immunity
Source: J Transl Med. 2024 May 30;22:519. doi: 10.1186/s12967-024-05331-3 (PMC11138045; doi:10.1186/s12967-024-05331-3)
Supplement: Supplementary file 8 — Supplementary Material 8 [file 12967_2024_5331_MOESM8_ESM.docx]

**Supplementary Table 1.** **Antibody information**

| **Antibodies** | **Source** | **Identifier** | **Application/Dilutions** |
| --- | --- | --- | --- |
| γ-H2AX | CST | #9718 | IF:1:1000 |
| Phospho-ATM (Ser1981) | CST | #5883 | WB:1:1000 |
| Phospho-ATR (Ser428) | CST | #2853 | WB:1:1000 |
| Phospho-Chk1 (Ser345) | CST | #2348 | WB:1:1000 |
| Phospho-Chk2 (Thr68) | CST | #2197 | WB:1:1000 |
| Phospho-DNA-PKcs (S2056) | Abcam | ab18192 | WB:1:2000 |
| GPX4(EPNCIR144) | Abcam | ab125066 | WB:1:5000 IHC:1:200 |
| Cox2 (D5H5) | CST | #12282 | WB:1:1000 |
| 4-HNE | Thermo Fisher | MA5-45790 | WB:1:1000 |
| SLC7A11(EPR27115-64) | Abcam | ab307601 | WB:1:1000 |
| Phospho-TBK1/NAK (Ser172) | CST | #5483 | WB:1:1000 |
| Phospho-IRF-3 (Ser396) | CST | #29047 | WB:1:1000 |
| IFN-β1 (D1D7G) | CST | #73671 | WB:1:1000 |
| STING | Abclonal | A20175 | WB:1:500 |
| Phospho-STING | Abclonal | AP1369 | WB:1:500 |
| IFN-β1 | Abclonal | A22740 | WB:1:2000 |
| GAPDH | CST | #5174 | WB:1:1000 |
| 8-OHDG | Bioss | bs-1278R | IF:1:200 |
| TOMM20 | Abcam | ab186735 | IF:1:250 |
| GM130 | BD | 610822 | IHC:1:300 |
| STING (D2P2F) | CST | #13647 | IHC:1:50 |
| Cleaved Caspase-3 | CST | #9664 | WB:1:1000 |
| Phospho-MLKL | CST | #18640 | WB:1:1000 |
| Phospho-RIP3 | CST | #93654 | WB:1:1000 |
| Zombie NIR Fixable Viability Kit | Biolegend | 423106 | 0.5ul/test |
| CD45 ‒ BV785 | Biolegend | 103149 | 1ul/test |
| CD45 ‒ BV510 | Biolegend | 103137 | 1ul/test |
| CD3-PE/Dazzle594 | Biolegend | 100245 | 1ul/test |
| CD4-FITC | Biolegend | 100406 | 1ul/test |
| CD8a-AF700 | Biolegend | 100730 | 1ul/test |
| CD62L-BV605 | Biolegend | 104437 | 1ul/test |
| PD-1-PE-cy7 | Biolegend | 135215 | 1ul/test |
| CD25-APC | Biolegend | 101910 | 1ul/test |
| CD44-Percp-cy5.5 | Biolegend | 103032 | 1.5ul/test |
| CD103-PE-cy7 | Biolegend | 121426 | 1.5ul/test |
| CD11C-AF488 | Biolegend | 117311 | 1.5ul/test |
| CD40-PE | Biolegend | 157506 | 1.5ul/test |
| MHCⅡ-BV605 | Biolegend | 107639 | 1.5ul/test |
| TIM-3-BV421 | Biolegend | 134019 | 2ul/test |
| CD80-APC | Biolegend | 104714 | 2ul/test |
| CD86-BV421 | Biolegend | 105032 | 2ul/test |
| GZMB-PE | Biolegend | 372208 | 2ul/test |
| IFN-γ-BV421 | Biolegend | 505829 | 2ul/test |
| FOXP3-PE | Biolegend | 126404 | 2.5ul/test |

**Supplementary Table 2. The primers used for quantitative real-time PCR analysis**

| **primers** | **sequence 5'-3'** |
| --- | --- |
| human-PTGS2-F | AAACTCTGGCTAGACAGCGTAA |
| human-PTGS2-R | AACCGTAGATGCTCAGGGAC |
| human-ACSL4-F | AGTACCCTGAAGGATTTGAGATT |
| human-ACSL4-R | TGGCCTGTCATTCCAGCT |
| human-SLC7A11-F | CGATACAAATGCCCAGAT |
| human-SLC7A11-R | CTCCGACATTATTCTAAACCA |
| human-GPX4-F | GCCGCCTTTGCCGCCTACTGAA |
| human-GPX4-R | CCATGTGCCCGTCGATGTCCTT |
| human-mtDNA-F | GTCTATTAATCTACCATCCTCCGTG |
| human-mtDNA-R | GATGAAAGTAGGCCAAAATAAAAAG |
| human-Ki67-F | GGGTCTGTTATTGATGAGCC |
| human-Ki67-R | GTTGACTTCCTTCCATTCTGA |
| human-GAPDH-F | AATCCCATCACCATCTTCCA |
| human-GAPDH-R | AAATGAGCCCCAGCCTTCT |
| mouse-IL-1b-F | TGTGTCTTTCCCGTGGACCT |
| mouse-IL-1b-R | TCGGAGCCTGTAGTGCAGTT |
| mouse-IL-6-F | CTTCTTGGGACTGATGCTGGT |
| mouse-IL-6-R | AGACAGGTCTGTTGGGAGTGG |
| mouse-IL-10-F | AGCTGTTTCCATTGGGGACA |
| mouse-IL-10-R | AAGTGTGGCCAGCCTTAGAAT |
| mouse-IL-12A-F | TTGCCCTCCTAAACCACCTC |
| mouse-IL-12A-R | GCCGTCTTCACCATGTCATC |
| mouse-IFN-γ-F | CAGGCCATCAGCAACAACATAA |
| mouse-IFN-γ-R | GGCAATACTCATGAATGCATCC |
| mouse-TNF-α-F | CCAACGGCATGGATCTCAAAG |
| mouse-TNF-α-R | ATAGCAAATCGGCTGACGGT |
| mouse-GAPDH-F | TGCAGTGGCAAAGTGGAGATT |
| mouse-GAPDH-R | GGCTTCCCGTTGATGACAAG |
